# The optimal operation decisions of expert service enterprises based on queuing system

**DOI:** 10.1371/journal.pone.0360850

**Published:** 2026-10-30

**Authors:** Jing Zhang, Lei Cui, Chen Liu, Minghui Jiang

**Affiliations:** 1 School of Economics and Management, East University of Heilongjiang, Harbin, Heilongjiang, China; 2 School of Management, Harbin Institute of Technology, Harbin, Heilongjiang, China; Wenzhou-Kean University, CHINA

## Abstract

Expert service enterprises constitute an important part of the service sector and play a significant role in its development. However, such enterprises face two major challenges. On the one hand, they must satisfy heterogeneous customer demand for experts of different ranks; on the other hand, they must manage compensation constraints in the expert labor market. To address these challenges, this study develops a queueing-theoretic framework that captures the joint effects of expert rank and service time. In doing so, it explicitly endogenies expert rank in the model and examines how enterprises optimize expert rank and service time under different demand scenarios. The results yield several key findings. First, the optimal expert rank chosen by the enterprise is independent of the overall demand volume, which is a somewhat unexpected result. This finding suggests that expert recruitment should be aligned with the firm’s long-term strategic positioning rather than adjusted merely in response to short-term demand fluctuations. Second, when market demand is strong, the enterprise can adopt a “low-margin, high-volume” strategy by jointly optimizing service time and service price, thereby expanding customer coverage and maximizing service throughput. Third, when demand is relatively low, the enterprise should carefully adjust service price and expert compensation to maintain profitability without compromising service quality. Overall, this study provides practical managerial insights for expert service enterprises seeking to optimize service delivery strategies, balance customer expectations with expert compensation, and avoid market distortions. By endogenizing expert rank and service time and analyzing equilibrium decision behavior, this study also extends the research perspective on expert service operations.

## 1. Introduction

Expert services are widely observed in consulting, healthcare, accounting, auditing, repair and maintenance, and other professional domains. Because these services rely heavily on providers’ knowledge, professional judgment, and specialized skills, they have become an increasingly important component of modern service economies [1]. From an industry-development perspective, the professional services market has also become a major segment of the global service economy. According to The Business Research Company, the global expert services market reached approximately USD 6.07 trillion in 2024 and is projected to increase to about USD 7.89 trillion by 2029, with a compound annual growth rate of approximately 5.39% [2]. This growth trend indicates that, as firms and individuals increasingly rely on specialized services such as consulting, healthcare, accounting, auditing, and repair and maintenance, the operational management of expert service enterprises is becoming increasingly important.

Unlike traditional services, expert services are characterized by high requirements for practitioners’ professional competence, customized and knowledge-intensive service delivery, relatively low dependence on physical capital, and a highly specialized workforce [3–5]. Firms in this sector typically employ skilled professionals to provide tailored services that satisfy heterogeneous customer needs. However, customers often prefer experts with higher professional credentials or stronger reputations, which may lead to congestion in high-tier expert queues. In contrast, lower-ranked experts may face insufficient demand, making it difficult for firms to fully utilize expert resources and maintain profitability. A typical example can be found in healthcare, where patients generally prefer to consult physicians with higher qualifications, while physicians with relatively lower credentials may be underutilized. Such an imbalance may not only reduce service accessibility and overall service efficiency but also result in inefficient allocation of professional resources [6]. Therefore, understanding how expert service enterprises make operational decisions in response to customers’ perceptions of expert status is both theoretically meaningful and practically important.

Although prior studies have examined expert services from different perspectives, several important research gaps remain. First, existing research has mainly focused on service quality, professional reputation, customer satisfaction, and knowledge-intensive service delivery [7–10]. However, relatively limited attention has been paid to how firms jointly determine expert rank, service time, and service price under customer queueing behavior. Second, studies on service operations and queueing systems have provided important insights into congestion management, pricing decisions, and capacity choices [11,12]. Nevertheless, these studies usually treat service capability as exogenous and do not explicitly capture the role of expert rank in shaping customer demand and waiting costs. Third, the expert labor market and the customer market have often been examined separately [13,14]. In practice, however, expert service enterprises must simultaneously attract customers and compensate experts. Hiring lower-ranked experts may reduce customers’ willingness to purchase services, whereas hiring higher-ranked experts may increase wage expenditures and impose cost pressure on the firm. These observations suggest that the interactions among customer demand, expert rank, service time, service pricing, and wage costs have not been sufficiently investigated within a unified analytical framework.

Accordingly, it is crucial for expert service enterprises to develop optimal service strategies that simultaneously address both customer and expert market dynamics. This study focuses on three core research questions:

- How can enterprises select experts of appropriate ranks, given customer preferences and wage structures in the expert market?
- How should service duration and pricing be optimized under varying levels of customer demand?
- How do optimal decisions and profit outcomes change across different scales of market demand?

To address these questions, we develop a customer utility function based on queuing theory to capture how expert rank, service duration, and pricing jointly influence customers’ service choices in expert service settings. Building on this utility framework, we formulate an enterprise-level optimization model to characterize firms’ optimal operational decisions under different demand scenarios. This modeling approach enables us to examine how expert service enterprises balance customer-side demand with expert-side compensation constraints, reduce potential market frictions, and improve overall profitability.

**The main contributions of this study are summarized as follows.**

- This study integrates the customer market and the expert labor market into a unified analytical framework. Unlike traditional service operation models that mainly focus on customer demand and waiting time, our model also considers expert remuneration constraints. This allows us to examine how enterprises balance customer-side service demand with expert-side compensation costs.
- This study characterizes the optimal operational decisions of expert service enterprises under different demand scenarios. Specifically, we analyze how enterprises should determine expert rank, service duration, and service price to improve operational efficiency and profitability.
- This study provides new managerial insights into expert service operations. One key finding is that the optimal expert rank remains unchanged regardless of the overall demand scale, suggesting that expert recruitment should be guided by long-term strategic positioning rather than short-term demand fluctuations. In high-demand markets, firms can improve profitability by adopting a “low-margin, high-volume” strategy, serving more customers through shorter service durations and lower prices.

## 2. Literature review

The research domains related to this paper mainly include expert services, optimal control, and queuing theory.

Expert services generally refer to a category of services provided by experts with professional knowledge and practical experience. Through diagnosis, evaluation, and professional judgment, experts provide corresponding support to customers in typical settings such as healthcare, accounting, auditing, maintenance, and consulting [4,15–18]. In these industries and organizations, how to effectively manage expert resources, improve expert service efficiency, and release the value of expert knowledge has become an important issue in service operations management. Existing studies have shown that experts’ professional judgments and proactive suggestions are important for both organizational operations and social development [1,19–21]. Due to the significant information asymmetry that usually exists between customers and experts, expert service markets may suffer from service inducement, whereby experts recommend or provide unnecessary services to customers in order to obtain higher returns [22]. This phenomenon has been verified in a large body of research [23–25]. Building on this stream of literature, Dai and Singh [6] further pointed out that reputational benefits may lead to a separating equilibrium between high-ranked and low-ranked experts. Du et al. [26] examined how consumer heterogeneity and expert pricing strategies affect platform information disclosure decisions and market outcomes in online expert service platforms. However, the existing literature has mainly focused on information asymmetry between experts and customers, service inducement, and platform information disclosure, while paying relatively limited attention to how consumer demand affects expert hiring decisions and operational behaviors of expert service enterprises. Therefore, this paper incorporates the expert labor market and the consumer market in expert services into a unified analytical framework, characterizes the interaction between the two markets, and extends expert service research from the perspective of the coordinated decision-making of expert hiring and service operations.

Optimal control methods have been widely applied in service operations, supply chain management, energy scheduling, intelligent transportation, and other fields. In recent years, researchers have extended optimal control methods from different application contexts. Zhao et al. [27] investigated a secure optimal control method for time-delay nonlinear systems based on multi-player Stackelberg-Nash games, providing new insights into control optimization in multi-agent dynamic decision-making environments. Brusset et al. [28] incorporated an epidemic dynamics model into the analysis of supply chain reconfiguration and ripple effects, and developed an optimal control model to optimize supply chain structures and reduce systemic risks. As energy and resource constraints have become increasingly prominent, operational optimization in the energy sector has also received considerable attention. Stejskal et al. [29] pointed out that improving the economic efficiency of power plants and energy enterprises through operational optimization is of great importance for the stable operation of energy systems and the efficient use of resources. Wu et al. [30] further focused on smart charging and analyzed the optimal strategy of delaying charging to periods with lower electricity prices. They designed a charging scheduling scheme that minimizes charging costs under endogenous and time-varying electricity procurement costs. In addition, with the development of artificial intelligence and intelligent optimization methods, optimal control has gradually expanded from traditional analytical models to data-driven and intelligent decision-making scenarios. For example, Li et al. [31] proposed a cross-domain intelligent traffic signal control method based on federated proximal policy optimization, aiming to optimize traffic signal control at typical intersections through distributed joint training across multiple domains. Similar intelligent optimization methods have also been applied and extended in various fields [32]. Overall, existing studies show that optimal control methods can effectively address dynamic decision-making, multi-agent interactions, and resource allocation problems in complex operational systems. However, the existing literature has paid relatively limited attention to the interrelationships among customer demand, expert rank, service duration, and pricing decisions in expert service enterprises. Different from the above studies, this paper simultaneously considers the customer market and the expert labor market within a queuing system framework, and analyzes the joint decision-making problem of expert rank, service duration, and service price, thereby further extending the application of optimization methods in service operations management.

This study is intrinsically linked to queuing theory. Debo et al. [22] pioneered the application of queuing theory to the analysis of expert services, suggesting that the phenomenon of service inducement is present and characterizing it through extended service durations. This paper, however, regards service duration as a crucial element influencing customer perceived utility. Anand et al. [33] delineated customer-intensive services based on service rates within queuing systems, examining optimal decisions regarding service rates. Following their work, Zhan et al. [34] explored optimal service strategies in customer-intensive services with a focus on the advertising effect. Yuan et al. [35] found that service providers could mitigate customer anxiety by providing entertainment options in waiting areas, integrating the concept of the waiting area entertainment into operational decision-making issues related to queuing theory. Zhou et al. [36], using taxi stands as a case, investigated how loss aversion affects queuing configurations in service queuing systems. Ma [37] addressed the challenge of minimizing the total idle time of vehicle charging by incorporating charger-level queuing delays under conditions of stochastic customer demand and charging congestion. However, there is a dearth of literature examining the influence of service duration on compensation decisions for experts, which means that the queuing characteristics within expert services are not fully represented. Consequently, this paper includes service duration as a decision variable for expert service enterprises and further analyzes the impact of changes in service duration on customer demand for expert ranks and compensation, thereby enriching the application of queuing theory in expert services.

Table 1 presents the differences between this study and previous studies.

**Table 1:** Comparison of this study with related literature.

Study	Queue	Utility	Pricing	Duration	Rank	Salary	Demand
Dai and Singh [6]	Yes	Yes	No	Yes	Yes	No	No
Debo et al. [22]	Yes	Yes	Yes	Yes	No	No	No
Anand et al. [33]	Yes	Yes	Yes	Yes	No	No	Yes
Zhan et al. [34]	Yes	Yes	Yes	Yes	No	No	Yes
Yuan et al. [35]	Yes	Yes	Yes	Yes	No	No	Yes
Gao and Su [38]	Yes	Yes	Yes	No	No	No	No
Zhan et al. [39]	Yes	Yes	Yes	No	No	No	No
Chung et al. [40]	No	No	No	No	Yes	Yes	No
**This study**	**Yes**	**Yes**	**Yes**	**Yes**	**Yes**	**Yes**	**Yes**

## 3. Model

### 3.1 Model assumptions

To clarify the analytical scope of the model, we first present the main assumptions underlying this study.

**Assumption 1. Expert service enterprises operate in two interconnected markets.** An expert service enterprise obtains customers from the customer market and hires experts from the expert labor market to provide services. The enterprise determines the expert rank, service duration, and service price to maximize its profit. This assumption reflects the operational feature of expert service enterprises, whose decisions are jointly constrained by customer demand and expert compensation [34].

**Assumption 2. Customers are sensitive to expert rank and service duration.** Customers evaluate expert services according to the basic perceived utility, expert rank, and service duration. The rank of expert is denoted by *r*, where a smaller *r* indicates a higher expert rank. The parameter *r*_0_ represents customers’ expected expert rank. When *r* < *r*_0_, customers obtain additional utility from a higher-ranked expert; when *r* > *r*_0_, customers perceive lower service utility. In addition, service duration affects customers’ perceived value because expert services usually require sufficient professional interaction and customized knowledge input.

**Assumption 3. Customers make joining decisions based on net utility.** Customers are rational and decide whether to enter the service system by comparing their perceived service utility with the service price and waiting cost. Specifically, customers join the system only when their net utility is non-negative. The waiting cost depends on the customer’s delay cost per unit of time and the system sojourn time [34,35]. This assumption provides the basis for deriving the effective arrival rate of customers under different demand conditions.

**Assumption 4. The service system follows an M/M/1 queueing structure.** Customers arrive according to a Poisson process, and service times follow an exponential distribution. The service discipline is First-Come, First-Served. Therefore, the expert service system can be modeled as an M/M/1 queue, which is widely used in service operations research [33,38,39]. The latent demand is denoted by Λ, and the effective customer arrival rate is denoted by λ, where 0≤λ≤Λ. The service duration is denoted by *t*, and the service rate is μ=1/t. To ensure system stability, we require 0≤λ<1/t.

**Assumption 5. Expert compensation increases with expert rank.** Experts with higher ranks require higher compensation. To capture the wage difference among experts within the same industry, the expert’s salary per unit of time is assumed to be a decreasing function of *r*. That is, a smaller *r* corresponds to a higher expert rank and therefore a higher salary. This assumption reflects the professional barriers and compensation differences in expert labor markets [40]. To avoid the unrealistic case in which the enterprise always hires the highest-ranked expert, we further assume that the wage disparity effect is sufficiently strong relative to customers’ sensitivity to expert rank.

### 3.2 Customer utility function

In expert services, the baseline perceived utility for customers is *V*_0_. We introduce the sensitivity coefficient ϵ as a measure of customers’ sensitivity to the rank of expert, reflecting their ability to discern the expert rank throughout the service. This sensitivity is also affected by the service duration *t*. As customers seek more detailed service, they can perceive grea*t*er utility within a certain range as *t* increases. Thus, the perceived utility func*t*ion for customers is modeled in this paper as follows:


$$\begin{array}{c} R(r,t)=V_{0}+\epsilon \left(r_{0}-r\right)t \end{array}$$ (1)

In Equation (1), customers are sensitive to the specific value of *r* within a given *t*, which is a characteristic of expert services. Equation (1) also indicates that, when *r* = *r*_0_, customers receive the basic utility *V*_0_. If *r* < *r*_0_, customers can derive additional utility from an increase in the rank of expert, hence *R*(*r*, *t*) > *V*_0_. Conversely, when $r>r_{0},R(r,t)<V_{0}$. This equa*t*ion implies that the utility of customers is influenced by $r,r_{0},\epsilon$, and *t*.

Table 2 presents Symbols and Definitions.

**Table 2:** Symbols and definitions.

Symbol	Definition
Λ	Arrival rate of potential customers (latent demand)
λ	Arrival rate of effective customers (effective demand)
*r*	Actual rank of expert
*r*_0_	Expected rank of expert
*V*_0_	Basic perceived utility
$V_{N}$	Net utility of service
*R*(*r*,*t*)	Perceived service utility
*P*	Service price
*t*	Service duration
*s*	Expert’s salary
α	Wage disparity within the industry
ϵ	Sensitivity to the rank of expert per unit of service time
*c*	Customer’s cost of delay per unit of time
*W*	System sojourn time

Furthermore, it is assumed that there exists a time threshold $\underset{―}{t}$, which signifies the shortest duration an expert requires to complete a service. If $t<\underset{―}{t}$, the expert is unable to finish the service; therefore, it is guaranteed that $t\geq \underset{―}{t}$, suggesting that *t* cannot tend towards zero indefinitely. The model by Debo et al. [22] contemplates that experts in services might u*t*ilize extended service durations to entice customers, suggesting that longer service times could be an attempt to provide superfluous services. In this paper, longer service durations are considered essential, such as when a psychiatrist devotes substantial time to monitoring a patient’s psychological shifts, thereby offering more effective treatment. We incorporate the effects of service duration, aligning the decision-making of expert service enterprises more closely with real-world operational circumstances.

The customer’s cost consists of two components: one is the service expense *P*, and the other is the delay cost *c W*. In this context, *c* signifies the customer’s cost per unit of delay, while *W* represents the system’s waiting time, also referred to as the sojourn time. Within the classical M/M/1 queuing model, the system’s waiting time is formulated as $W(\lambda ,t)=\frac{1}{\mu -\lambda }=\frac{t}{1-\lambda t}$, with the system’s stability condition being $0\leq \lambda <\frac{1}{t}$; thus, the customer’s delay cost is calculated as $cW(\lambda ,t)=\frac{ct}{1-\lambda t}$. Therefore, the customer’s net utility is articulated as follows:


$$\begin{array}{c} V_{N}=V_{0}+\epsilon \left(r_{0}-r\right)t-P-cW(\lambda ,t)=R(r,t)-P-cW(\lambda ,t). \end{array}$$ (2)

(1) The scenario where $R(r,t)-P-\frac{ct}{1-\Lambda t}\geq 0$. $\frac{ct}{1-\Lambda t}$ represents the waiting cost for all customers entering the service system, indicating that in this scenario, the demand is relatively low, and any customer entering the system can obtain a positive utility. In this case, the effective customer arrival rate (effective market demand) is λ=Λ.
(2) The scenario where $ct\leq R(r,t)-P<\frac{ct}{1-\Lambda t}$. *ct* is the waiting cost when there are no other customers in the system, suggesting that in this scenario, the demand is relatively high, and only some customers entering the system can achieve non-negative utility. The equilibrium condition for this scenario is $R(r,t)-P-\frac{ct}{1-\lambda t}=0$, hence the effective customer arrival rate is $\lambda =\frac{1}{t}-\frac{c}{R(r,t)-P}$.
(3) The scenario where R(r,t)−P<ct. The net utility obtained by customers is always negative, and no customers will join the system, resulting in an effective customer arrival rate of λ=0.

Compared with traditional studies, such as Yuan et al. [35] and Zhan et al. [41], which treat the utility generated by expert rank and service time as exogenous, this study makes several contributions. First, we endogenize expert rank and service time in the model, examine how they jointly affect customer utility, and further derive the optimal expert rank and service duration. Second, we integrate the customer market and the expert labor market into a unified analytical framework, thereby capturing the interaction between customer demand, expert compensation, and firm operational decisions. Finally, we analyze firm decisions under different demand scenarios and derive meaningful managerial insights for expert service enterprises.

### 3.3 Demand segmentation and threshold derivation

The customer’s cost consists of two components: the service price *P* and the delay cost *cW*. Here, *c* denotes the customer’s delay cost per unit of time, and *W* denotes the system sojourn time. In the classical M/M/1 queueing model, the system sojourn time is given by


$$\begin{array}{c} W(\lambda ,t)=\frac{1}{\mu -\lambda }=\frac{t}{1-\lambda t}, \end{array}$$ (3)

where μ=1/t is the service rate and 0≤λ<1/t is the stability condition. Therefore, the customer’s delay cost is $cW(\lambda ,t)=\frac{ct}{1-\lambda t}$. Thus, the customer’s net utility can be written as


$$\begin{array}{c} V_{N}=V_{0}+\epsilon (r_{0}-r)t-P-cW(\lambda ,t)=R(r,t)-P-\frac{ct}{1-\lambda t}. \end{array}$$ (4)

For notational simplicity, let A(P,r,t)=R(r,t)−P, where *A*(*P*,*r*,*t*) represents the customer’s perceived service u*t*ility net of the service price but before considering the waiting cost. Thus, the customer’s net utility can be rewritten as


$$\begin{array}{c} V_{N}(\lambda ;P,r,t)=A(P,r,t)-\frac{ct}{1-\lambda t}. \end{array}$$ (5)

Since the waiting cost $\frac{ct}{1-\lambda t}$ increases with the effective arrival rate λ, the customer’s net utility decreases as more customers enter the service system. Therefore, the effective arrival rate is determined by whether customers can still obtain non-negative utility under different levels of congestion. Based on this logic, customers’ joining behavior can be divided into the following three cases.

First, when $A(P,r,t)\geq \frac{ct}{1-\Lambda t}$, even if all potential customers enter the service system, each customer can still obtain non-negative net utility. In other words, the service system can accommodate the entire potential demand without violating the customers’ participation condition. Therefore, all potential customers join the system, and the effective arrival rate is λ=Λ. This case corresponds to an insufficient-demand market, because the latent demand does not exceed the maximum demand that the service system can sustain under the given service policy.

Second, when $ct\leq A(P,r,t)<\frac{ct}{1-\Lambda t}$, customers can obtain non-negative utility when congestion is sufficiently low, because A(P,r,t)≥ct implies that the service is attractive even when there is only one customer in the system. However, if all potential customers enter the system, the resulting congestion would make customers’ net utility negative, since $A(P,r,t)<\frac{ct}{1-\Lambda t}$. Hence, only part of the potential demand enters the service system in equilibrium. The equilibrium effective arrival rate is determined by the zero-net-utility condition:


$$\begin{array}{c} A(P,r,t)-\frac{ct}{1-\lambda t}=0. \end{array}$$ (6)

Solving this equation yields


$$\begin{array}{c} \lambda =\frac{1}{t}-\frac{c}{A(P,r,t)}=\frac{1}{t}-\frac{c}{R(r,t)-P}. \end{array}$$ (7)

This case corresponds to a sufficient-demand market, because potential demand is high enough that congestion, rather than lack of customers, determines the realized effective demand.

Third, when *A*(*P*,*r*,*t*) < *ct*, even if there is no congestion, the customer’s net utility is negative because the minimum delay cost is *ct*. Therefore, no customer joins the service system, and the effective arrival rate is λ=0.

Combining the above three cases, the effective customer arrival rate can be expressed as


$$\begin{array}{c} \lambda (P,r,t)=\left\{ \begin{array}{cc} \Lambda , & \text{if }0\leq P\leq R(r,t)-\frac{ct}{1-\Lambda t},\\ \frac{1}{t}-\frac{c}{R(r,t)-P}, & \text{if }R(r,t)-\frac{ct}{1-\Lambda t}<P\leq R(r,t)-ct,\\ 0, & \text{if }P>R(r,t)-ct. \end{array}\right. \end{array}$$ (8)

The above segmentation shows that the effective demand depends on whether the latent demand Λ can be fully accommodated while ensuring non-negative customer utility. For a given expert rank *r* and service duration *t*, the demand threshold $\hat{\lambda }(P,r,t)$ represents *t*he maximum effective arrival rate that can be sustained in equilibrium. When the latent demand is below this threshold, all potential customers enter the system. When the latent demand exceeds this threshold, only part of the potential customers enter the system, and the effective demand is determined by the zero-net-utility condition.

Accordingly, for any given service policy (*P*,*r*,*t*), the demand threshold can be defined as


$$\begin{array}{c} \hat{\lambda }(P,r,t)=\frac{1}{t}-\frac{c}{R(r,t)-P}. \end{array}$$ (9)

This threshold represents the maximum effective arrival rate that can be sustained while ensuring that customers obtain non-negative net utility. In other words, when the effective arrival rate exceeds $\hat{\lambda }(P,r,t)$, the waiting cost becomes sufficiently high that customers’ net utility turns negative, and some potential customers will choose not to enter the service system.

When $0\leq \Lambda \leq \hat{\lambda }(P,r,t)$, the potential market demand does not exceed the maximum sustainable arrival rate. Therefore, all potential customers are willing to enter the service system, and the effective arrival rate is λ=Λ. This case is classified as an insufficient-demand market, because the service system is not constrained by congestion but by the limited size of potential demand.

When $\Lambda >\hat{\lambda }(P,r,t)$, the potential market demand exceeds the maximum sustainable arrival rate. In this case, if all potential customers entered the system, congestion would make customers’ net utility negative. Therefore, only part of the potential customers enter the service system in equilibrium, and the effective arrival rate is determined endogenously by the zero-net-utility condition. This case is classified as a sufficient-demand market, because the potential demand is large enough, and the realized demand is limited by congestion and customers’ participation constraints rather than by the size of the market.

### 3.4 Enterprise’s profit function

Let us consider an expert service enterprise that seeks to maximize profits. This enterprise provides services to customers by hiring experts and charges fees to generate revenue. The enterprise also needs to pay salaries to the experts, with the compensation determined based on the rank of expert. Chung et al. [40] argue that expert services have high barriers to entry, and experts within a particular industry often find it challenging to work in other industries. Therefore, this paper only considers the impact of income differences within the industry on expert salaries to capture the characteristic of high professional barriers inherent in expert services. Thus, this paper presents the expert’s salary *s* per unit of time as follows:


$$\begin{array}{c} s=\frac{\alpha }{1+r}. \end{array}$$ (10)

Equation (10) indicates that the expert’s salary *s* increases as the rank of expert rises (i.e., as *r* decreases), reaching its maximum level α when the expert rank is at its peak (*r* = 0). α represents the wage disparity between the highest and lowest ranks of expert within the industry; it is also essential that α>ϵ to ensure that the service enterprise does not indefinitely raise the rank of expert. Based on this, the profit function of the enterprise can be expressed as follows:


$$\begin{array}{c} \pi (P,r,t)=(P-st)\lambda (P,r,t). \end{array}$$ (11)

In Equation (11), P≥st, implies that the unit net profit is positive; otherwise, the enterprise will exit the market. To achieve the optimal profit, the enterprise must ensure that $V_{N}\geq 0$. Particularly when the rank of experts reaches its highest level (*r* = 0), the customer utility must be non-negative, that is, $V_{0}+\epsilon r_{0}t-ct-\alpha t\geq 0$, to ensure that there are customers in the system at this time.

### 3.5 Demand scale segmentation

As can be inferred from above section, the scale of demand will influence the equilibrium state of the service system. Additionally, Anand et al. [33] have delineated the developmental stages of service enterprises based on the scale of demand, highlighting the necessity of categorizing demand. Based on customer utility, the effective customer arrival rate (market demand) function can be derived as follows:


$$\begin{array}{c} \lambda (P,r,t)=\left\{ \begin{array}{cc} \Lambda & \text{ if }0\leq P\leq R-\frac{ct}{1-\Lambda t},\\ \frac{1}{t}-\frac{c}{R-P} & \text{ if }R-\frac{ct}{1-\Lambda t}<P\leq R-ct,\\ 0 & \text{ if }R-ct<P. \end{array}\right. \end{array}$$ (12)

Based on the profit function outlined in Equation (11), optimizing the price yields the optimal pricing for a given rank of experts *r* and service duration *t* as follows:


$$\begin{array}{c} P^{*}(r,t)=\left\{ \begin{array}{cc} R-\frac{ct}{1-\Lambda t} & \text{ if }0\leq \Lambda \leq \hat{\lambda },\\ R-\sqrt{ct(R-st)} & \text{ if }\Lambda >\hat{\lambda }. \end{array}\right. \end{array}$$ (13)

The threshold $\hat{\lambda }$ represents the maximum number of customers that the service provider can accommodate in given *r* and *t*. $0\leq \Lambda \leq \hat{\lambda }$ indicates insufficient demand, meaning tha*t* the latent demand has not reached the service system’s capacity limit, allowing all customers to enter the system. Conversely, $\Lambda >\hat{\lambda }$ signifies sufficient demand, where only a number of customers equivalent to $\hat{\lambda }$ can be served, and the rest will exit. At this point, λ is the effective customer arrival rate when demand is abundant and the service price is $P^{*}(r,t)$. Therefore, the expression for $\hat{\lambda }$ is as follows:


$$\begin{array}{c} \hat{\lambda }\left(P^{*}(r,t),r,t\right)=\lambda_{suf}^{*}\left(P^{*}(r,t),r,t\right)=\frac{1}{t}-\sqrt{\frac{c}{t(R-st)}}. \end{array}$$ (14)

Based on the threshold $\hat{\lambda }$ mentioned above, this paper categorizes the market into markets with insufficient demand ($0\leq \Lambda \leq \hat{\lambda }$) and markets with sufficient demand (Λ> $\hat{\lambda }$), and investigates *t*, *r*, and $P^{*}(r,t)$ under different demand scales.

## 4. Decision analysis with insufficient demand

### 4.1 Optimal decision making

In situations where demand is insufficient, $0\leq \Lambda \leq \hat{\lambda }$, all customers can be served, hence the effective customer arrival rate is defined as Λ. According to Equation (6), when $0\leq \Lambda \leq \hat{\lambda }$, the optimal pricing strategy under given *r* and *t* is expressed as $P_{ins}^{*}(r,t)=$ $R-\frac{ct}{1-\Lambda t}$. Based on this, we can derive the profit function for the service enterprise as follows:


$$\begin{array}{c} \pi_{ins}\left(P_{ins}^{*},r,t\right)=\left(P_{ins}^{*}-st\right)\lambda \left(P_{ins}^{*},r,t\right)=\left(R-\frac{ct}{1-\Lambda t}-\frac{\alpha t}{1+r}\right)\Lambda ,\text{ when }0\leq \Lambda \leq \hat{\lambda }. \end{array}$$ (15)

Given that $\frac{\partial \pi_{ins}\left(P_{ins}^{*}r,t\right)}{\partial r}=\left(\frac{\alpha t}{(1+r{)}^{2}}-\epsilon t\right)\Lambda ,\frac{\partial^{2}\pi_{ins}\left(P_{ins}^{*}r,t\right)}{\partial r^{2}}=-2\alpha \Lambda t(1+r{)}^{-3}<0$, and we can deduce the following proposition.

**Proposition 1.** *Since* α>ϵ*, there must exist an optimal expert rank* $r_{ins}^{*}=\sqrt{\frac{\alpha }{\epsilon }}-1$ *that ensures the enterprise achieves maximum profit. Consequently, the optimal effective customer arrival rate* $\lambda_{ins}^{*}\left(P^{*},r^{*},t\right)$*, service pricing* $P_{ins}^{*}\left(r^{*},t\right)$*, expert salary* $s_{ins}^{*}\left(r^{*},t\right)$*, and the enterprise profit* $\pi_{ins}^{*}\left(P^{*},r^{*},t\right)$ *can be expressed as follows: (Note: “ins” denotes the scenario of insufficient demand scale).*


$$\begin{array}{c} \left\{ \begin{array}{c} P_{ins}^{*}\left(r^{*},t\right)=V_{0}+\epsilon \left(r_{0}+1\right)t-t\sqrt{\epsilon \alpha }-\frac{ct}{1-\Lambda t^{\prime }},\\ s_{ins}^{*}\left(r^{*},t\right)=\sqrt{\epsilon \alpha },\\ \lambda_{ins}^{*}\left(P^{*},r^{*},t\right)=\Lambda ,\\ \pi_{ins}^{*}\left(P^{*},r^{*},t\right)=\left(V_{0}+\epsilon \left(r_{0}+1\right)t-2t\sqrt{\epsilon \alpha }-\frac{ct}{1-\Lambda t}\right)\Lambda . \end{array}\right. \end{array}$$ (16)

Proposition 1 demonstrates that, under given conditions to time *t* and in scenarios of insufficient demand scale, the optimal rank of expert $r_{ins}^{*}$ is exclusively influenced by the industry’s wage differential α and customer sensitivity ϵ. When $r_{ins}\in \left(0,\sqrt{\frac{\alpha }{\epsilon }}-1\right)$, it follows that $\frac{\partial \pi_{ins}\left(P^{*},r,t\right)}{\partial r}>0$, indicating that reducing the rank of expert can improve the enterprise’s optimal profit when the expert rank is high, and the optimal profit is attained when $r_{ins}^{*}=\sqrt{\frac{\alpha }{\epsilon }}-1$. Nevertheless, if a service enterprise continues to lower its expert rank, it must substantially reduce its prices to ensure that the customer’s perceived utility remains non-negative, which is not conducive to profit generation.

Furthermore, it is discernible that the service duration *t* does not play a role in the establishment of the rank of expert. Service enterprises, when engaging experts, predominantly take into account the market dynamics of the expert market (characterized by the industry’s wage differential, α) and the customer market (the sensitivity of customers to the expert rank, ϵ). The service duration *t*, as an element influencing the service process, does not alter the hiring practices of service enterprises in *t*he labor market. Nevertheless, alterations in *t* can have repercussions on the delay costs experienced by customers within the service system, as well as on the profi*t*ability of the enterprise.

In Proposition 1, it is further observed that $\frac{\partial r_{ins}^{*}}{\partial \epsilon }<0$, and $\frac{\partial^{2}r_{ins}^{*}}{\partial \epsilon^{2}}>0$. This indicates that $r_{ins}^{*}$ will decrease as ϵ increases, with the rate of decrease tending to slow down over time. An increase in ϵ signifies that customers are increasingly sensitive to the expert rank, which would lead service enterprises to engage experts at a higher rank (a lower $r_{ins}^{*})$ to satisfy customer needs. However, the fact that the rate of decrease in $r_{ins}^{*}$ is diminishing suggests that the inclination of service enterprises to hire experts at higher ranks is waning, as higher-rank experts typically demand higher salaries. This also underscores that the improvement in the rank of expert is subject to the limitation imposed by the salary $s_{ins}^{*}=\sqrt{\epsilon \alpha }$.

From the analysis provided, the optimal profit is expressed as $\pi_{ins}^{*}\left(P^{*},r^{*},t\right)=$ $\left(V_{0}+\epsilon \left(r_{0}+1\right)t-2t\sqrt{\epsilon \alpha }-\frac{ct}{1-\Lambda t}\right)\Lambda$. By calculating the first and second derivatives of $\pi_{ins}^{*}\left(P^{*},r^{*},t\right)$ with respect to *t*, we can have:


$$\begin{array}{c} \left\{ \begin{array}{c} \frac{\partial \pi_{ins}^{*}\left(P^{*},r^{*},t\right)}{\partial t}=\left(\epsilon \left(r_{0}+1\right)-2\sqrt{\epsilon \alpha }-\frac{c}{(1-\Lambda t{)}^{2}}\right)\Lambda ,\\ \frac{\partial^{2}\pi_{ins}^{*}\left(P^{*},r^{*},t\right)}{\partial t^{2}}=\frac{-2c\Lambda }{(1-\Lambda t{)}^{3}}<0. \end{array}\right. \end{array}$$ (17)

**Proposition 2.** *There exists an optimal service duration* $t_{\text{ins }}^{*}=\frac{1}{\Lambda }\left(1-\sqrt{\frac{c}{\epsilon \left(r_{0}+1\right)-2\sqrt{\epsilon \alpha }}}\right)$ *that maximizes the profit* $\pi_{\text{ins }}^{*}\left(P_{\text{ins }}^{*},r_{\text{ins }}^{*},t\right)$*. Consequently, the optimal decisions for the service provider and the corresponding optimal profit can be represented as follows:*


$$\begin{array}{c} \left\{ \begin{array}{c} P_{ins}^{*}\left(r_{ins}^{*},t_{ins}^{*}\right)=V_{0}+\epsilon \left(r_{0}+1\right)t_{ins}^{*}-t_{ins}^{*}\sqrt{\epsilon \alpha }-\frac{ct_{ins}^{*}}{1-\Lambda t_{ins}^{*}},\\ s_{ins}^{*}\left(r_{ins}^{*},t_{ins}^{*}\right)=\sqrt{\epsilon \alpha },\\ \lambda_{ins}^{*}\left(P_{ins}^{*},r_{ins}^{*},t_{ins}^{*}\right)=\Lambda ,\\ \pi_{ins}^{*}\left(P_{ins}^{*},r_{ins}^{*},t_{ins}^{*}\right)=\Lambda \left(V_{0}+\epsilon \left(r_{0}+1\right)t_{ins}^{*}-2t_{ins}^{*}\sqrt{\epsilon \alpha }-\frac{ct_{ins}^{*}}{1-\Lambda t_{ins}^{*}}\right). \end{array}\right. \end{array}$$ (18)

Proposition 2 shows that $t_{ins}^{*}$ is influenced by *r*_0_, which represents customers’ expected expert rank required to complete the service and also reflects the baseline complexity of the service. An increase in *r*_0_ indicates a lower requirement for expert rank, implying that the service is less difficult to complete. Therefore, the positive relationship between $t_{ins}^{*}$ and *r*_0_ suggests that when customers’ expected expert rank decreases, expert service enterprises may extend the service duration to enhance customers’ perceived utility. In addition, $t_{ins}^{*}$ is sensitive to changes in potential demand Λ. As Λ increases, $t_{ins}^{*}$ decreases, indicating that when the customer base expands, firms should shorten the service duration to serve more customers. Overall, Proposition 2 suggests that expert service enterprises facing insufficient demand can determine their optimal service strategy and achieve maximum profit by conducting appropriate market research and calibrating key market parameters.

### 4.2 Sensitivity analysis

From the analysis presented, it is evident that the optimal service duration is defined as $t_{\text{ins }}^{*}=\frac{1}{\Lambda }\left(1-\sqrt{\frac{c}{\epsilon \left(r_{0}+1\right)-2\sqrt{\epsilon \alpha }}}\right)$. The derivative of $t_{\text{ins }}^{*}$ with respect to ϵ can be calculated as follows:


$$\begin{array}{c} \frac{\partial t_{ins}^{*}}{\partial \epsilon }=\frac{c}{2\Lambda }{\left(\frac{c}{\epsilon \left(r_{0}+1\right)-2\sqrt{\epsilon \alpha }}\right)}^{-\frac{1}{2}}\left(\frac{\left(r_{0}+1\right)-\sqrt{\frac{\alpha }{\epsilon }}}{{\left(\epsilon \left(r_{0}+1\right)-2\sqrt{\epsilon \alpha }\right)}^{2}}\right). \end{array}$$ (19)

**Corollary 1.** *When* $0\leq \Lambda \leq \hat{\lambda }$*, there exists a threshold* $\epsilon_{m}=\max\left(\frac{4\alpha }{{\left(r_{0}+1\right)}^{2}},\frac{\sqrt{\alpha }+\sqrt{\alpha +c\left(r_{0}+1\right)}}{r_{0}+1}\right)$*. As* ϵ *increases within the interval* $\epsilon \in \left(\epsilon_{m},\alpha \right),t_{\text{ins }}^{*}$ *also increases. However, when* ϵ *is within the range* $\epsilon \in \left(0,\epsilon_{m}\right]$*, such customers are no longer the target customers for the service enterprise, and subsequent analyses will not consider these customers.*

Corollary 1 indicates that when the customer’s sensitivity $\epsilon \in \left(\epsilon_{m},\alpha \right)$, the optimal service duration $t_{ins}^{*}$ increases as ϵ increases. However, when $\epsilon \in \left(0,\epsilon_{m}\right]$, due to the low customer sensitivity, these customers are not considered the target audience for the enterprise, and experts will not provide services to such customers. The lower bound of sensitivity $\epsilon_{m}$ effectively determines the target customers. When $\epsilon \leq \epsilon_{m}$, it suggests that customers are less sensitive to the rank of expert, and in situations of insufficient demand, these low-sensitivity customers prevent service enterprises from achieving higher profits by setting higher prices (as high prices are likely to result in $V_{N}<0$). Therefore, service enterprises will not offer services to such customers. In reality, these customers are also not keen on seeking expert services, exhibiting a distrust towards experts. For instance, some patients, when ill, do not wish to seek medical treatment, believing they can recover on their own.

When customer sensitivity exceeds the threshold $\epsilon_{m}$, that is, when $\epsilon \in (\epsilon_{m},\alpha )$, customers become more responsive to expert rank. As ϵ increases, customers derive greater utility from higher-ranked experts, which enables the enterprise to charge higher prices and obtain greater profits. To further enhance customers’ perceived utility, the enterprise may encourage experts to extend the service duration and thereby capture additional profit. In this case, the benefit generated by the increase in customer utility outweighs the additional cost of longer service time, such as higher expert compensation. Therefore, as customer sensitivity ϵ increases within the interval $\left(\epsilon_{m},\alpha \right)$, the optimal strategy is to extend the service duration for each customer, thereby improving customers’ perceived utility and increasing the profitability.

Based on the characteristics of the target customers established in Corollary 1, the impact of customer sensitivity on $P_{ins}^{*}$ and $\pi_{ins}^{*}$ can be analyzed.

**Corollary 2.** *Given that in the scenario of insufficient demand, the target customers satisfy* $\epsilon \in \left(\epsilon_{m},\alpha \right)$*, and* $\epsilon_{m}=\max\left(\frac{4\alpha }{{\left(r_{0}+1\right)}^{2}},\frac{\sqrt{\alpha }+\sqrt{\alpha +c\left(r_{0}+1\right)}}{r_{0}+1}\right)$*. It follows that:*

i. *When* $0\leq \Lambda \leq \hat{\lambda }$*, the effective demand* $\lambda_{\text{ins }}^{*}$ *is unaffected by* ϵ;
ii. $P_{\text{ins }}^{*}\left(r_{\text{ins }}^{*},t_{\text{ins }}^{*}\right)$ *will monotonically increase with* ϵ *within the range* $\left(\epsilon_{m},\alpha \right)$;
iii. $\pi_{\text{ins }}^{*}\left(P_{\text{ins }}^{*},r_{\text{ins }}^{*},t_{\text{ins }}^{*}\right)$ *will monotonically increase with* ϵ *within the range* $\left(\epsilon_{m},\alpha \right)$.

Corollary 2 indicates that under the condition of $0\leq \Lambda \leq \hat{\lambda }$, both the service enterprise’s $P_{ins}^{*}\left(r_{ins}^{*},t_{ins}^{*}\right)$ and $\pi_{ins}^{*}\left(P_{ins}^{*},r_{ins}^{*},t_{ins}^{*}\right)$ monotonically increase with ϵ within the range ($\epsilon_{m},\alpha$). Analysis based on $r_{ins}^{*}$ and $t_{ins}^{*}$ reveals that within this range, an increase in ϵ leads to a decrease in $r_{ins}^{*}$ and an increase in $t_{ins}^{*}$, meaning that not only is the rank of expert enhanced, but also the service duration is extended. This significantly improves the perceived utility for customers during the expert service process; it also results in increased expenditures for the service enterprise on expert salaries. Consequently, the service enterprise should raise the price $P_{ins}^{*}$ to offset the increased costs, thereby effectively enhancing the service enterprise’s profit $\pi_{ins}^{*}$. This implies that target customers who are more sensitive to the rank of expert can prompt the service enterprise to provide higher quality expert services, and higher quality expert services also bring higher profits to the enterprise.

## 5. Decision analysis with insufficient demand

### 5.1 Optimal decision making

In markets with sufficient demand, where $\Lambda >\hat{\lambda }$, only a portion of customers can achieve non-negative utility and enter the service system. Based on Equations 13 and 14, the optimal pricing is given by $P_{\text{suf }}^{*}(r,t)=R(r)-\sqrt{ct(R(r,t)-st)}$, and the effective arrival rate is $\lambda_{\text{suf }}\left(P^{*},r,t\right)=\frac{1}{t}-\sqrt{\frac{c}{t(R(r,t)-st{)}^{\prime }}}$, leading to the profit function as follows:


$$\begin{array}{cc} \pi_{suf}\left(P_{suf}^{*},r,t\right) & =(R(r)-st-\sqrt{ct(R(r)-st)})\left(\frac{1}{t}-\sqrt{\frac{c}{t(R(r)-st)}}\right)\\ & ={\left(\sqrt{\frac{R(r)-st}{t}}-\sqrt{c}\right)}^{2}. \end{array}$$ (20)

It can be deduced that $\frac{\partial \pi_{suf}\left(P_{suf}^{*},r,t\right)}{\partial r}=\frac{1}{t}\left(1-\sqrt{\frac{ct}{V_{0}+\epsilon \left(r_{0}-r\right)t-\frac{\alpha t}{1+r}}}\right)\left(\frac{\alpha t}{(1+r{)}^{2}}-\epsilon t\right)$. Moreover, since P≥st, it follows that $V_{0}+\epsilon \left(r_{0}-r\right)t-\frac{\alpha t}{1+r}ct\geq ct$, hence the sign of $\frac{\partial \pi_{suf}\left(P_{suf}^{*},r,t\right)}{\partial r}$ is determined by $\frac{\alpha }{(1+r{)}^{2}}-\epsilon$.

**Proposition 3.** *When* $\Lambda >\hat{\lambda }$*, there exists an optimal rank of expert* $r_{\text{suf }}^{*}=\sqrt{\frac{\alpha }{\epsilon }}-1$ *that maximizes* $\pi_{\text{suf }}\left(P_{\text{suf }}^{*},r,t\right)$*. Moreover,* $r_{\text{suf }}^{*}=r_{\text{ins }}^{*}$. Concurrently, the optimal effective customer arrival rate $\lambda_{\text{suf }}^{*}\left(P_{\text{suf }}^{*},r_{\text{suf }}^{*},t\right)$*, service pricing* $P_{\text{suf }}^{*}\left(r_{\text{suf }}^{*},t\right)$*, expert salary* $s_{\text{suf }}^{*}\left(r_{\text{suf }}^{*},t\right)$*, and enterprise profit* $\pi_{\text{suf }}^{*}\left(P_{\text{suf }}^{*},r_{\text{suf }}^{*},t\right)$ *can be expressed as follows (Note: “suf” denotes the scenario of sufficient market demand):*


$$\begin{array}{c} \left\{ \begin{array}{c} P_{suf}^{*}\left(r_{suf}^{*},t\right)=V_{0}+\epsilon \left(r_{0}+1\right)t-t\sqrt{\epsilon \alpha }-\sqrt{ct\left(V_{0}+\epsilon \left(r_{0}+1\right)t-2t\sqrt{\epsilon \alpha }\right)},\\ s_{suf}^{*}\left(r_{suf}^{*},t\right)=\sqrt{\epsilon \alpha },\\ \lambda_{suf}^{*}\left(P_{suf}^{*},r_{suf}^{*},t\right)=\frac{1}{t}-\sqrt{\frac{c}{t\left(V_{0}+\epsilon \left(r_{0}+1\right)t-2t\sqrt{\epsilon \alpha }\right)}},\\ \pi_{suf}^{*}\left(P_{suf}^{*},r_{suf}^{*},t\right)={\left(\sqrt{\frac{\left(V_{0}+\epsilon \left(r_{0}+1\right)t-2t\sqrt{\epsilon \alpha }\right)}{t}}-\sqrt{c}\right)}^{2}. \end{array}\right. \end{array}$$ (21)

Proposition 3 illustrates that when $\Lambda >\hat{\lambda }$, the determination of $r_{suf}^{*}$ is still solely dependent on α and ϵ, which is identical to $r_{ins}^{*}$ in Proposition 1, that is, $r_{suf}^{*}=r_{ins}^{*}$. However, unlike Proposition 1, Proposition 3 indicates that under this circumstance, $\lambda_{suf}^{*}$ is no longer a constant value. Therefore, when formulating optimal decisions, it is necessary to consider the impact of the variation in $\lambda_{suf}^{*}$ on profit. Additionally, the service duration *t* also affects the specific value of $\lambda_{suf}^{*}$, making the optimal service duration a point of interest.

Regarding service duration *t*, completing an expert service requires a certain amount of time and effort from the expert. Without loss of generality, we assume that experts of different ranks differ only in the quality of the services they provide, which is a key characteristic of expert services, while the minimum service duration $\underset{―}{t}$ required to complete the service is the same across expert ranks. Although the minimum service duration may vary in practice, such variation does not affect the main conclusions of our analysis.

From Proposition 3, it is known that the optimal profit for a service enterprise with sufficient demand is given by $\pi_{\text{suf }}^{*}\left(P_{\text{suf }}^{*},r_{\text{suf }}^{*},t\right)={\left(\sqrt{\frac{\left(V_{0}+\epsilon \left(r_{0}+1\right)t-2t\sqrt{\epsilon \alpha }\right)}{t}}-\sqrt{c}\right)}^{2}$. Taking the first-order derivative with respect to *t* yields:


$$\begin{array}{c} \frac{\partial \pi_{suf}^{*}}{\partial t}=-(T-\sqrt{c})(T{)}^{-\frac{1}{2}}\left(\frac{V_{0}}{t^{2}}\right)<0, \end{array}$$ (22)

where $T=\sqrt{\frac{\left(V_{0}+\epsilon \left(r_{0}+1\right)t-2t\sqrt{\epsilon \alpha }\right)}{t}}$. From the above equation, it can be inferred that $\pi_{suf}^{*}$ decreases as *t* increases. Therefore, shorter service durations can lead to higher profits for the enterprise, but i*t* must be recognized that the service duration cannot approach zero. Hence, the following proposition exists.

**Proposition 4.** *There exists an optimal service duration* $t_{suf}^{*}=\underset{―}{t}$ *that maximizes* $\pi_{\text{suf }}^{*}\left(P_{\text{suf }}^{*},r_{\text{suf }}^{*},t\right).\underset{―}{t}$ *represents the shortest duration required for an expert to complete an expert service. Thus, the final optimal decision and profit for the service enterprise can be expressed as follows:*


$$\begin{array}{c} \left\{ \begin{array}{c} P_{suf}^{*}\left(r_{suf}^{*},t_{suf}^{*}\right)=V_{0}+\epsilon \left(r_{0}+1\right)\underset{―}{t}-\underset{―}{t}\sqrt{\epsilon \alpha }-\sqrt{ct\left(V_{0}+\epsilon \left(r_{0}+1\right)\underset{―}{t}-2\underset{―}{t}\sqrt{\epsilon \alpha }\right)},\\ s_{suf}^{*}\left(r_{suf}^{*},t_{suf}^{*}\right)=\sqrt{\epsilon \alpha },\\ \lambda_{suf}^{*}\left(P_{suf}^{*},r_{suf}^{*},t_{suf}^{*}\right)=\frac{1}{\frac{t}{t}}-\sqrt{\frac{c}{\underset{―}{t}\left(V_{0}+\epsilon \left(r_{0}+1\right)\underset{―}{t}-2t\sqrt{\epsilon \alpha }\right)}},\\ \pi_{suf}^{*}\left(P_{suf}^{*},r_{suf}^{*},t_{suf}^{*}\right)={\left(\sqrt{\frac{\left(V_{0}+\epsilon \left(r_{0}+1\right)\underset{―}{t}-2\underset{―}{t}\sqrt{\epsilon \alpha }\right)}{\underset{―}{t}}}-\sqrt{c}\right)}^{2}. \end{array}\right. \end{array}$$ (23)

In the scenario where $\Lambda >\hat{\lambda }$, service enterprises can formulate optimal decisions and achieve the corresponding optimal profits based on the aforementioned propositions. Of course, service enterprises also need to pay attention to the characteristics of target customers when market demand is sufficient. Ensuring that $P_{suf}^{*}$ and $\lambda_{suf}^{*}$ are non-negative, it can be derived that target customers should meet the condition $c<\frac{V_{0}}{\underset{―}{t}}+\epsilon \left(r_{0}+1\right)-$ $2\sqrt{\epsilon \alpha }=\bar{c}$. This indicates that the unit delay cost for target customers should not be too high; otherwise, it will be difficult for service enterprises to provide services that yield a positive net utility for customers. At the same time, the larger the basic utility *V*_0_ or the shorter the minimum service duration $\underset{―}{t}$, the greater the allowable range of the unit delay cost *c* for target customers, which is more conducive to customer entry into the system. Fig 1 illustrates the distribution of target customers in a market with sufficient demand, from which it can be observed that as customer sensitivity ϵ increases, the allowable range of the unit delay cost for target customers expands, meaning that more customers will be able to become target customers. The parameter settings in Fig 1 are $V_{0}=40,\underset{―}{t}=1,\alpha =2,r_{0}=1$. The units of the relevant parameters are provided in Section 6.

**Fig 1:**
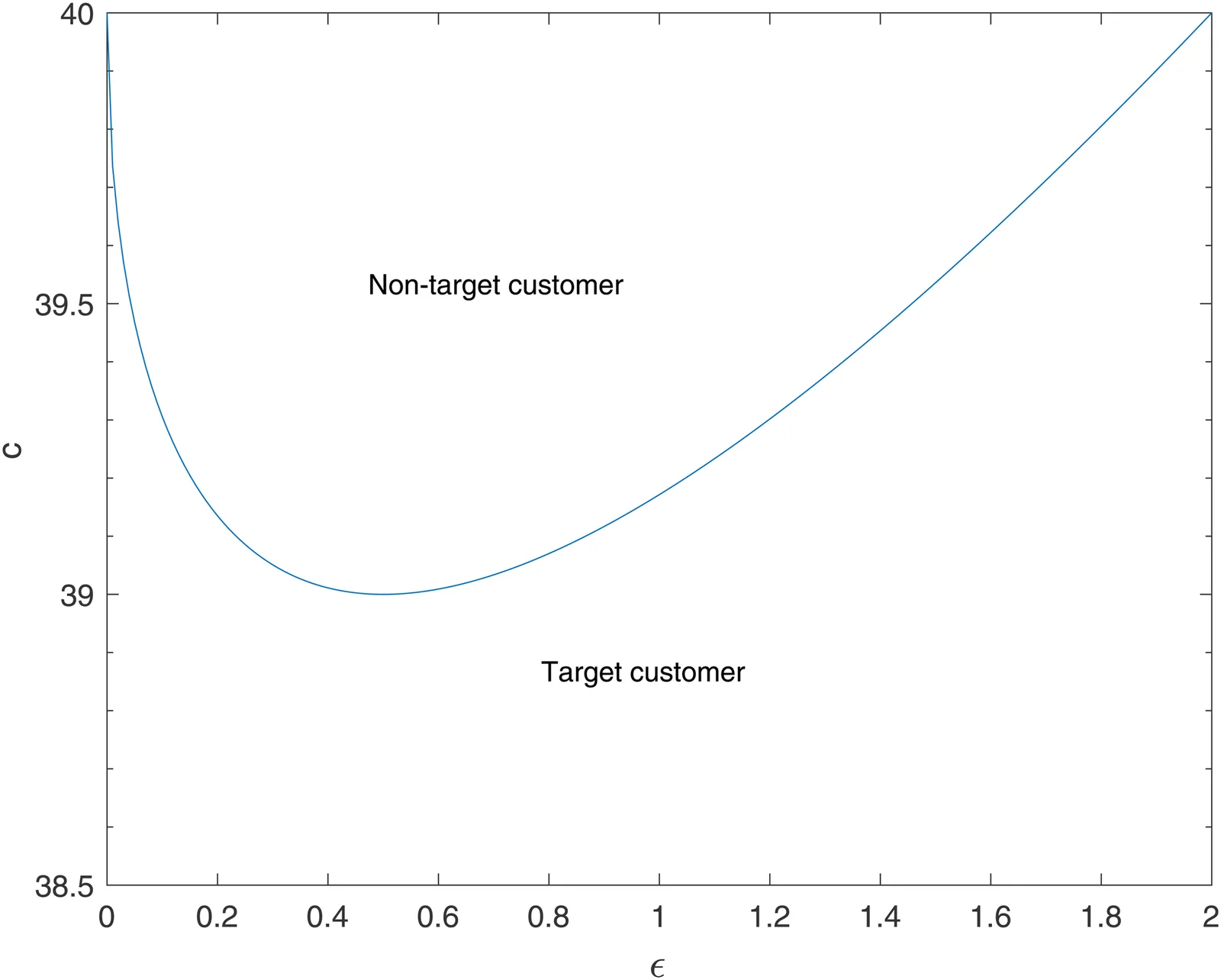
Distribution of target customers.

The optimal service duration $t_{suf}^{*}$ should be equal to the shortest duration $\underset{―}{t}$ that an expert can complete the service because when $\Lambda >\hat{\lambda }$, the potential customer arrival rate is high. Prolonging $t_{suf}^{*}$ would not only lead to congestion in the service system but also prevent serving more customers within a given time frame. This is detrimental to the profit acquisition of the service enterprise. Therefore, service enterprises must require experts to complete expert services as quickly as possible, which results in the optimal duration $t_{suf}^{*}$ for each expert to complete the expert service being the shortest duration $\underset{―}{t}$. This is significantly different from the optimal service duration in the scenario where 0≤ $\Lambda \leq \hat{\lambda }$.

When $\Lambda >\hat{\lambda }$, the potential market demand exceeds the maximum number of customers that the service system can accommodate, giving the service enterprise a market advantage. In this case, the enterprise does not need to further increase customer utility to attract demand. Instead, it should encourage experts to accelerate the service process so that more customers can be served within a given period. For example, in medical services, when patient demand is relatively low, doctors can spend more time on basic diagnosis and communication, thereby showing greater concern for patients and enhancing their perceived utility. However, during busy periods when hospitals face a large number of patients, doctors may have to make quick diagnoses and have limited time to address patients’ emotional needs. This mismatch between patient expectations and available service time may also contribute to tensions between doctors and patients.

### 5.2 Sensitivity analysis

The derivatives of $P_{suf}^{*}\left(r_{suf}^{*},t_{suf}^{*}\right),\lambda_{suf}^{*}\left(P_{suf}^{*},r_{suf}^{*},t_{suf}^{*}\right)$, and $\pi_{suf}^{*}\left(P_{suf}^{*},r_{suf}^{*},t_{suf}^{*}\right)$ with respect to ϵ can be obtained as follows:


$$\begin{array}{c} \left\{ \begin{array}{c} \frac{\partial P_{suf}^{*}}{\partial \epsilon }=\underset{―}{t}\left(\left(1-\sqrt{\frac{c\underset{―}{t}}{V_{0}+\epsilon \left(r_{0}+1\right)\underset{―}{t}-2\underset{―}{t}\sqrt{\epsilon \alpha }}}\right)\left(\left(r_{0}+1\right)-\sqrt{\frac{\alpha }{\epsilon }}\right)+\frac{1}{2}\sqrt{\frac{\alpha }{\epsilon }}\right),\\ \frac{\partial \lambda_{suf}^{*}}{\partial \epsilon }=\frac{\underset{―}{t}\sqrt{c}}{2{\left(V_{0}+\epsilon \left(r_{0}+1\right)\underset{―}{t}-2\underset{―}{t}\sqrt{\epsilon \alpha }\right)}^{\frac{3}{2}}}\left(\left(r_{0}+1\right)\underset{―}{t}-\underset{―}{t}\sqrt{\frac{\alpha }{\epsilon }}\right),\\ \frac{\partial \pi_{suf}^{*}}{\partial \epsilon }=\frac{1}{\underset{―}{t}}\left(1-\sqrt{\frac{c\underset{―}{t}}{V_{0}+\epsilon \left(r_{0}+1\right)\underset{―}{t}-2\underset{―}{t}\sqrt{\epsilon \alpha }}}\right)\left(\left(r_{0}+1\right)\underset{―}{t}-\underset{―}{t}\sqrt{\frac{\alpha }{\epsilon }}\right). \end{array}\right. \end{array}$$ (24)

**Corollary 3.** *There exists a critical value* $\bar{c}=\frac{V_{0}}{\underset{―}{t}}+\epsilon \left(r_{0}+1\right)-2\sqrt{\epsilon \alpha }$*, within the range* ϵ∈ $(0,\alpha ],\lim_{c\to \bar{c}}\frac{\partial P_{suf}^{*}}{\partial \epsilon }>0$*, indicating that* $P_{suf}^{*}$ *increases as* ϵ *increases. When c approaches 0, there must exist a critical value* $\bar{\epsilon }=\max_{\epsilon \in \left(0,\frac{\alpha }{{\left(r_{0}+1\right)}^{2}}\right)}\left\{\epsilon |\;\frac{\partial P_{suf}^{*}}{\partial \epsilon }=0\right\}$*, where for* $(0,\bar{\epsilon }],\frac{\partial P_{suf}^{*}}{\partial \epsilon }\leq 0;$ *and for* $\epsilon \in (\bar{\epsilon },\alpha ],\frac{\partial P_{suf}^{*}}{\partial \epsilon }>0$*.*

**Corollary 4.** *When* $\epsilon \in \left(0,\frac{\alpha }{{\left(r_{0}+1\right)}^{2}}\right)$*, the optimal effective customer arrival rate* $\lambda_{suf}^{*}$ *and the enterprise’s profit* $\pi_{suf}^{*}$ *will decrease as* ϵ *increases; whereas when* $\epsilon \in \left[\frac{\alpha }{{\left(r_{0}+1\right)}^{2}},\alpha \right],\lambda_{suf}^{*}$ *and* $\pi_{suf}^{*}$ *will increase as* ϵ *increases.*

When customer sensitivity is high, that is, $\epsilon \in \left[\frac{\alpha }{{\left(r_{0}+1\right)}^{2}},\alpha \right]$. As ϵ increases, customers can perceive a higher utility from the improvement in the expert rank, and the increased perceived utility will outweigh the costs incurred by the customers. Customers will be more willing to purchase expert services, which leads to an increase in the effective arrival rate. Moreover, according to the changes in $r_{suf}^{*}$ and $s_{suf}^{*}$ as indicated in Proposition 4, the increase in ϵ raises the rank and the salary of experts. This means that while ϵ enhances customer utility, it also increases the operating costs of the service enterprise. Therefore, the service enterprise should correspondingly raise the optimal price $P_{suf}^{*}$ to compress customer net utility and compensate for its own costs.

When $\epsilon \in \left(0,\frac{\alpha }{{\left(r_{0}+1\right)}^{2}}\right)$, the market consists of customers with low sensitivity. Based on the changes in $r_{suf}^{*}$, an increase in ϵ will prompt the service enterprise to hire experts at a higher rank. However, contrary to expectations, a higher rank of expert leads to a lower effective customer arrival rate. The reason for this is not a decrease in customers’ willingness to purchase services, but rather a limitation in service capacity. In this scenario, there is an upper limit to the number of customers the service enterprise can serve, denoted as the threshold $\hat{\lambda }$. In a market with sufficient demand, $\hat{\lambda }=\lambda_{suf}=\frac{1}{t}-\sqrt{\frac{c}{t(R(r,t)-st)}}$, where the increase in ϵ that leads to a higher rank of expert increases customer perceived utility, but also increases the salary of experts $s_{suf}^{*}$. Moreover, since $\epsilon \in \left(0,\frac{\alpha }{{\left(r_{0}+1\right)}^{2}}\right)$, the increase in expert salary is more significant compared to the increase in customer utility, leading to a decrease in the upper limit of the number of customers the service system can serve, $\hat{\lambda }$. At the same time, this indicates that although customers’ willingness to purchase increases, the decrease in the threshold $\hat{\lambda }$ will limit the increase in the effective customer arrival rate. That is, in an expert service market where supply is short of demand, the quantity of supply determines the quantity of demand. The decrease in $\hat{\lambda }$ will also lead to a reduction in the service enterprise’s profit $\pi_{suf}^{*}$.

Regarding the changes in the optimal price $P_{suf}^{*}$, it is influenced by the unit delay costc. When *c* tends towards $\frac{V_{0}}{\underset{―}{t}}+\epsilon \left(r_{0}+1\right)-2\sqrt{\epsilon \alpha },P_{suf}^{*}$ will increase with the increase of ϵ within the range ϵ∈(0,α]. However, when *c* tends towards $0,P_{suf}^{*}$ will first decrease and then increase with ϵ. Yet, due to the limitation of $\hat{\lambda }$, this ultimately cannot prevent the trend of the service enterprise’s profit from decreasing in this scenario. The following figure illustrates the variation of $P_{suf}^{*}$ with ϵ under different values of *c*. The parameters in the Fig 2 are set as $V_{0}=40,\underset{―}{t}=1,\alpha =2$, and *r*_0_ = 1. The units of the relevant parameters are provided in Section 6.

**Fig 2:**
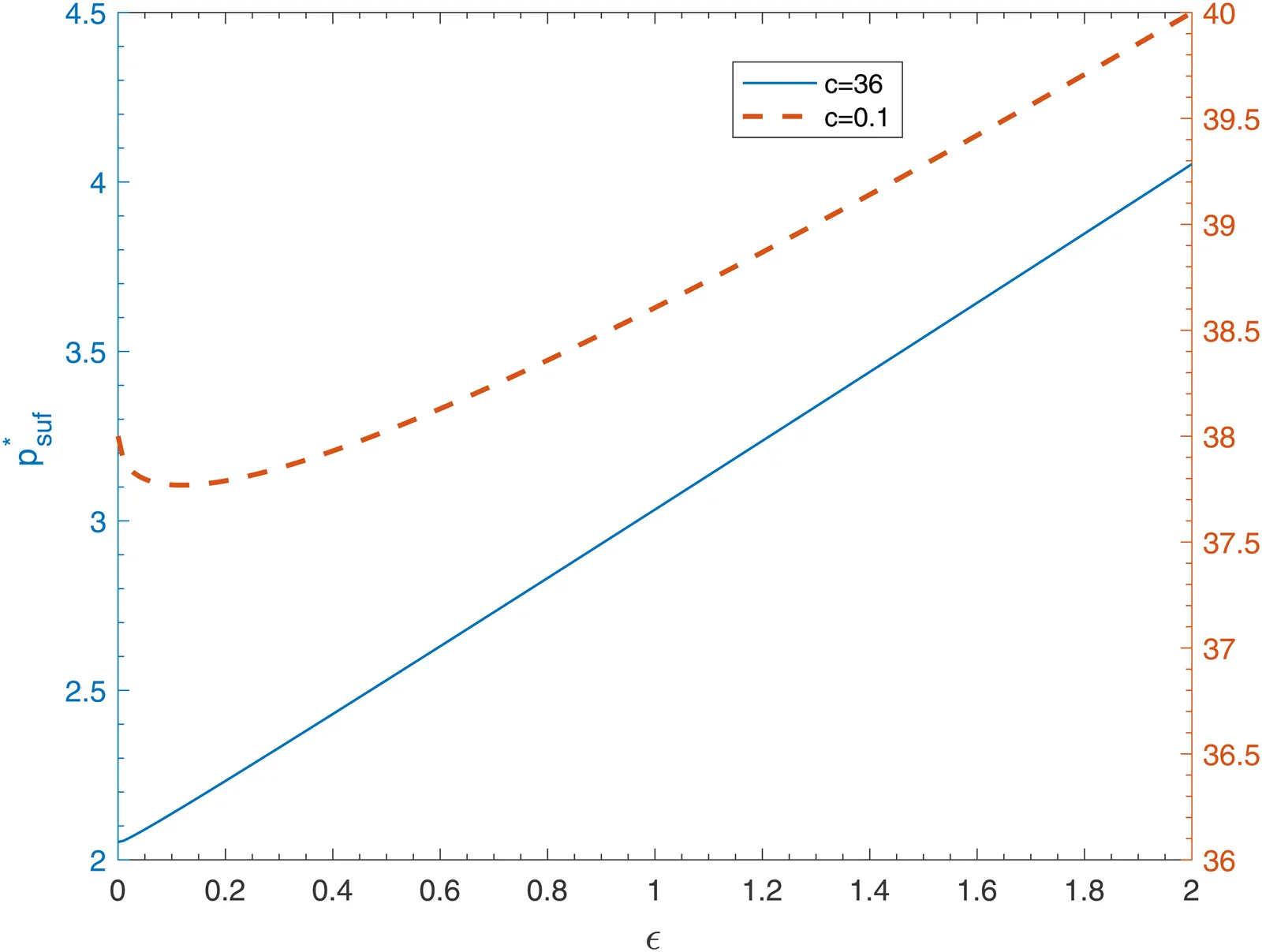
Variation of $P_{suf}^{*}$ with ϵ under different values of *c.*

Fig 2 above indicates that when the unit delay cost *c* for customers is relatively small, the service pricing $P_{suf}^{*}$ decreases and then increases with ϵ, exhibiting a concave function-like change. However, this decreasing trend is short-lived, only appearing when ϵ is small. When *c* is larger, $P_{suf}^{*}$ continues to rise with ϵ. Furthermore, it can be observed that when *c* is smaller, the $P_{suf}^{*}$ that the enterprise can set is significantly higher than in the case where *c* is larger. This implies that a reduction in customers’ delay costs will be beneficial for the enterprise to increase its service pricing.

### 5.3 Comparative analysis

From the analysis above, it can be inferred that when demand is insufficient (0≤Λ≤ $\hat{\lambda }$), the target customers should satisfy $\epsilon \in \left(\epsilon_{m},\alpha \right)$, where $\epsilon_{m}=$ $\max\left(\frac{4\alpha }{{\left(r_{0}+1\right)}^{2}},\frac{\sqrt{\alpha }+\sqrt{\alpha +c\left(r_{0}+1\right)}}{r_{0}+1}\right)$; and when demand is sufficient ($\Lambda >\hat{\lambda }$), the target customers of the enterprise should have a unit delay cost *c* and sensitivity ϵ that satisfy c∈ $\left(0,\frac{V_{0}}{t}+\epsilon \left(r_{0}+1\right)-2\sqrt{\epsilon \alpha }\right)$. Under the condition of $0\leq \Lambda \leq \hat{\lambda }$, every customer can obtain positive utility, so the service enterprise does not need to consider the situation where customer utility might be negative.

At the same time, $\epsilon_{m}$ will be influenced by $\alpha ,r_{0}$, and *c*. Only when the customer’s sensitivity ϵ is higher than $\epsilon_{m}$, can the service enterprise make a profit by providing services to such customers. In the case of $\Lambda >\Lambda >\hat{\lambda }$, not all customers can be served, and in the equilibrium state, $V_{N}=0$. Therefore, the service enterprise has requirements for the relationship between *c* and ϵ to ensure that when there are more people in the system, customer utility is non-negative. Moreover, *V*_0_ and $\underset{―}{t}$ also affect the range of intervals that *c* satisfies, the larger *V*_0_ is, or the smaller $\underset{―}{t}$ is, can increase the range of intervals for c. After comparing the optimal decisions, effective customer arrival rates, and enterprise profits under two different demand scales, the following corollaries can be derived.

**Corollary 5.** *Comparing markets with sufficient demand and insufficient demand, it is observed that: i.* $r_{ins}^{*}=r_{suf}^{*},t_{ins}^{*}>t_{suf}^{*}=\underset{―}{t},P_{ins}^{*}>P_{suf}^{*};ii.\lambda_{ins}^{*}\leq \lambda_{suf}^{*},\pi_{ins}^{*}<\pi_{suf}^{*}$.

Corollary 5 indicates that the optimal expert rank selected by the service enterprise remains the same regardless of whether market demand is sufficient or insufficient. This finding has important implications for expert service enterprises. When hiring experts, enterprises need to consider both the demand side, represented by customers’ sensitivity to expert rank ϵ, and the supply side, represented by the salary differentiation among experts α. The optimal expert rank therefore reflects an equilibrium within the “customer demand–expert rank–expert compensation” system. This result suggests that, regardless of market demand conditions, service enterprises can determine the appropriate expert rank directly based on α and ϵ, thereby reducing the complexity of their hiring and operational decisions.

The result $P_{ins}^{*}>P_{suf}^{*}$ indicates that the optimal price is higher under insufficient demand than under sufficient demand. This is because, when $0\leq \Lambda \leq \hat{\lambda }$, service enterprises cannot generate additional profit by increasing the number of customers and therefore need to raise the charge per customer to improve profitability. Although the profit obtained under this strategy is lower than $\pi_{suf}^{*}$ when demand is sufficient, it remains the optimal strategy under insufficient demand.

Combining the results $t_{ins}^{*}>t_{suf}^{*}$, $P_{ins}^{*}>P_{suf}^{*}$, and $\lambda_{ins}^{*}\leq \lambda_{suf}^{*}$, we can infer that when market demand is sufficient, service enterprises should adopt a “low-margin, high-volume” service strategy. Specifically, given that $r_{ins}^{*}=r_{suf}^{*}$, enterprises should encourage experts to serve as many customers as possible by adopting the shortest feasible service duration, namely $t_{suf}^{*}=\underset{―}{t}$. Since the service duration is shortened, the enterprise should charge a lower price $P_{suf}^{*}$ to prevent customers from leaving the system. Ultimately, the enterprise can obtain higher profits by serving a larger customer base. This “low-margin, high-volume” strategy enables expert service enterprises facing sufficient demand to capture a larger market share and achieve higher profitability.

### 5.4 Scalability and computational complexity

The proposed analytical framework is also computationally tractable and scalable. Since the main optimal decisions, including expert rank, service duration, service price, effective demand, and firm profit, are derived in closed form or through threshold-based analytical conditions, the model does not require high-dimensional numerical optimization or iterative search procedures for each decision instance. For a given set of parameters, the firm can determine the applicable demand scenario and then directly compute the corresponding optimal decisions. Therefore, the computational complexity of solving one decision instance is *O*(1).

When the framework is applied to multiple markets, service branches, expert categories, or customer segments, the computation can be repeated independently for each scenario. If there are *N* independent scenarios, the overall computational complexity increases linearly as *O*(*N*). Similarly, in numerical experiments or sensitivity analyses, if a parameter grid contains *G* combinations, the computational burden is *O*(*G*), since each grid point only requires direct substitution into the analytical expressions. This feature makes the proposed method suitable for large-scale scenario analysis and practical decision support.

In addition, if expert ranks are discrete in practice, the firm can evaluate a finite set of candidate ranks and select the one that yields the highest profit. If there are *K* candidate expert ranks, the corresponding enumeration complexity is *O*(*K*) for each market scenario, which remains computationally efficient for practical applications. Therefore, although the model is developed as an analytical framework, it can be extended to larger operational settings involving multiple service units, multiple expert categories, or heterogeneous market environments without imposing substantial computational burden.

## 6. Numerical simulation analysis

To enhance the interpretability of the numerical analysis, we assign explicit units to all parameters and interpret the simulation results as an illustrative numerical experiment rather than an empirical validation. Following the numerical approach of Anand et al. [33], time-related variables are measured in hours, demand is measured as the number of customers per hour, and monetary variables, including price, wage cost, customer utility, waiting cost, and firm profit, are measured in monetary units (MUs). Customer utility is expressed in monetary-equivalent terms so that it can be directly compared with service price and waiting cost. In the benchmark setting, we consider a healthcare service context. The salary differentiation coefficient across expert ranks is set to α=7.7 MUs per expert-rank level. The base utility that a patient obtains from receiving the service is set to *V*_0_ = 40 MUs per visit. The minimum service duration required for a doctor to complete a treatment is set to $\underset{―}{t}=0.1$ hours, which corresponds to approximately 6 minutes. The reference expert rank is normalized to *r*_0_ = 1. The patient waiting-cost parameter is set to *c* = 6 MUs per hour, which is equivalent to 0.1 MUs per minute. The potential market demand is set to Λ=5 patients per hour. Under this parameterization, the stability condition of the queueing system is satisfied in the numerical experiments, ensuring that the expected waiting time is well defined. Based on these benchmark values, we examine how the optimal price, effective demand, and firm profit vary under insufficient-demand and sufficient-demand scenarios. The price changes are reported in Fig 3, the changes in effective demand are shown in Fig 4, and the changes in firm profit are presented in Fig 5. To avoid overinterpreting a single parameter setting, we further conduct sensitivity analyses by varying the key parameters, including the salary differentiation coefficient, base customer utility, potential demand, and customer waiting-cost parameter. These analyses allow us to examine whether the main qualitative findings remain robust under different operational environments.

**Fig 3:**
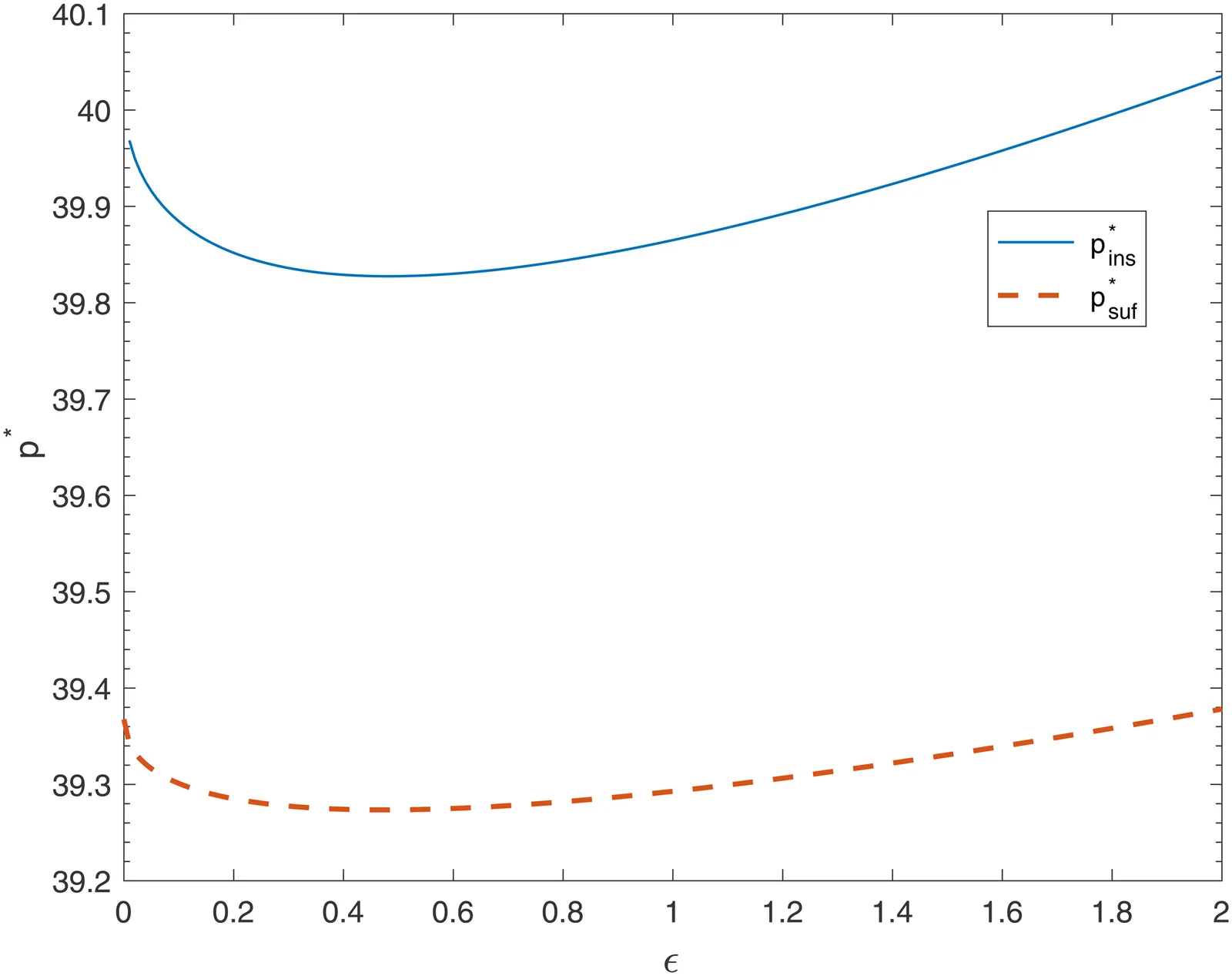
Optimal price variation with respect to ϵ.

**Fig 4:**
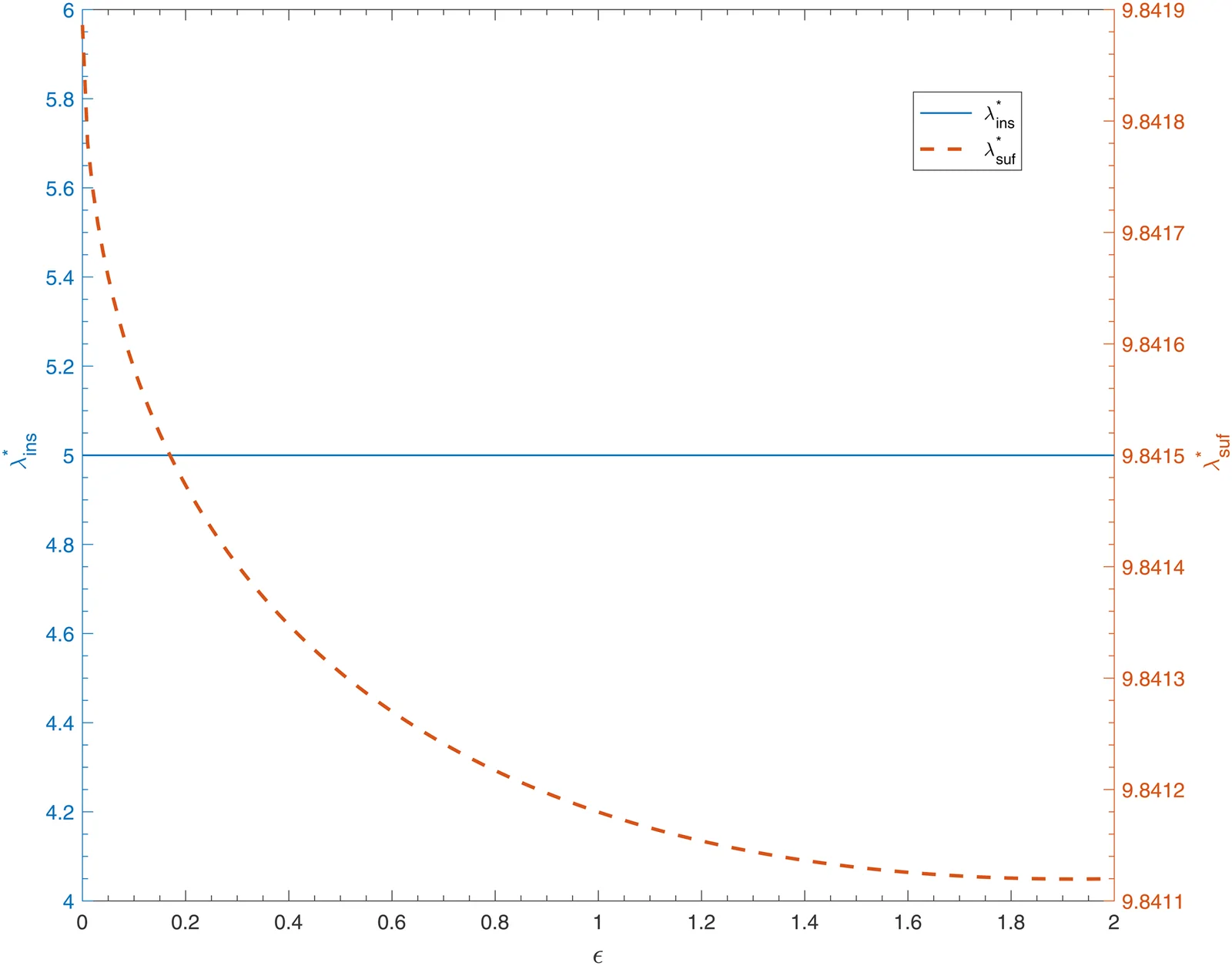
Optimal effective demand variation with respect to ϵ.

**Fig 5:**
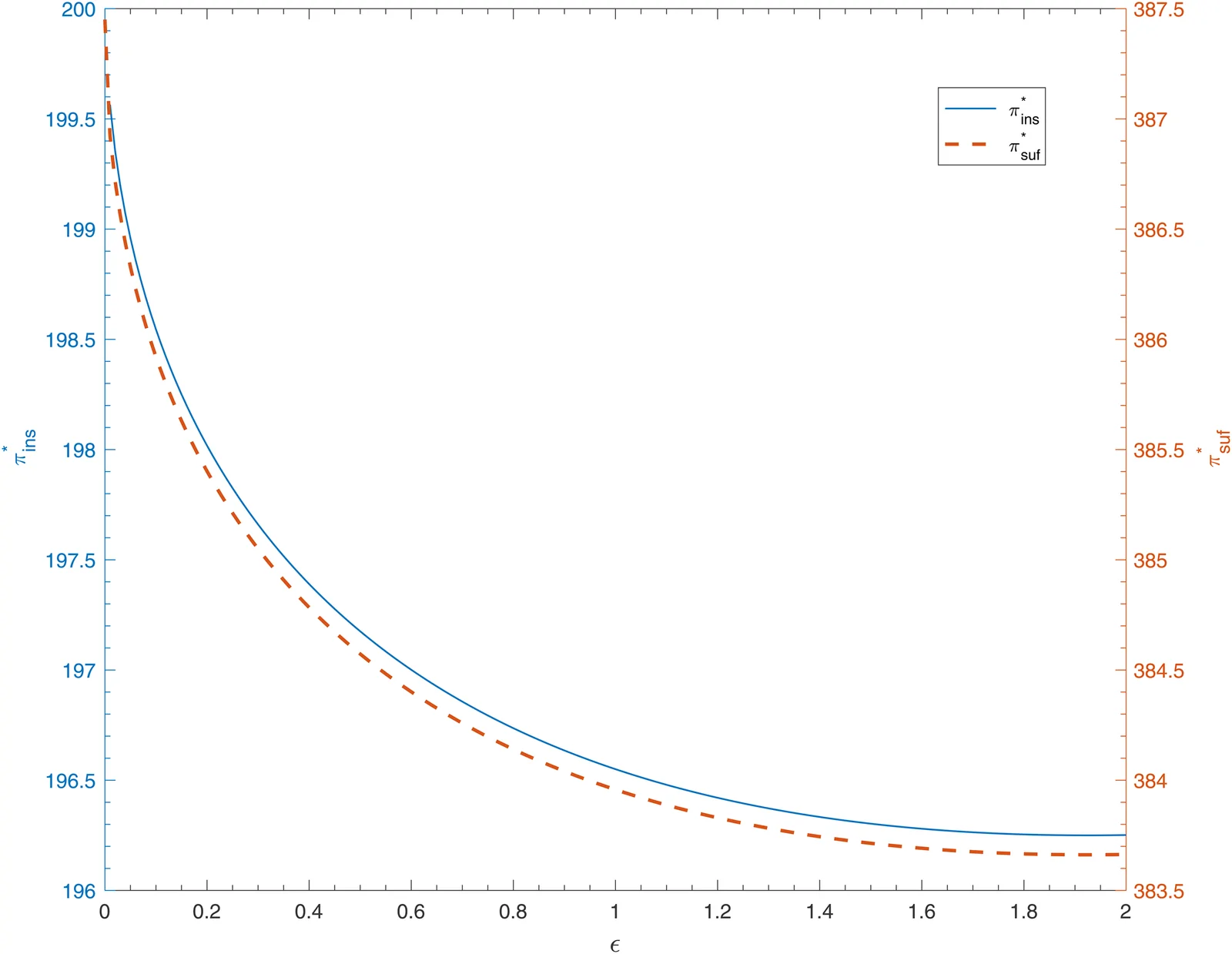
Optimal profit variation with respect to ϵ.

The purpose of the numerical analysis is not to claim empirical validation of the model, but to illustrate the economic mechanisms derived from the analytical results and to show how the proposed framework can be applied to different expert service contexts. In practice, expert service enterprises may calibrate the model using industry-specific data, such as wage differences across expert ranks, customers’ baseline service valuation, service completion times, market arrival rates, and customers’ delay costs. With such calibrated data, the model can support more context-specific decisions regarding service pricing, service duration, and expert-rank selection.

Fig 3 indicates that the price under conditions of insufficient demand is higher than that under conditions of sufficient demand, corroborating the results of Corollary 5. It also demonstrates that in both scenarios of insufficient and sufficient demand, the optimal price in the medical industry initially decreases and then increases with the rise of ϵ. This implies that when the sensitivity ϵ of patients to the doctor’s rank varies at a higher level, hospitals must raise their pricing to compensate for the investment in medical costs.

Fig 4 shows that as the patient sensitivity ϵ increases, the effective demand under conditions of insufficient demand remains constant, since the number of patients the hospital can serve is limited to the total number of patients present in the market. When there is sufficient demand in the market, the number of patients the hospital can treat depends on the service capacity limit of the hospital at that time, which decreases as the importance of the doctor’s rank to patients, ϵ, increases.

Fig 5 illustrates that as the importance of the doctor’s rank to patients, ϵ, increases, the actual profit that hospitals can obtain will correspondingly decrease. This is because hospitals must hire higher-rank doctors to provide treatment for patients, which to some extent raises the operational costs of the hospital, leading to reduced profits. In conjunction with Proposition 4, it can be understood that this is due to the high level of salary differentiation within the medical service industry, α, and the low level of patient sensitivity, ϵ, at this time. Therefore, regardless of whether the demand is sufficient or insufficient, the hospital’s profit will decrease as ϵ increases.

## 7. Conclusion

### 7.1 Results

This paper develops an expert service system based on the M/M/1 queueing model to investigate the optimal operational decisions of expert service enterprises. In the proposed framework, customers generate demand according to their perceived utility of expert services, while expert service enterprises hire experts from the expert labor market to provide services and obtain profits. By incorporating expert rank, service duration, service price, waiting cost, and expert salary into a unified analytical framework, this study examines how enterprises balance customer demand and expert compensation under different demand scales. Based on theoretical modeling and numerical analysis, the main results are summarized as follows.

(1) The optimal expert rank selected by the enterprise is the same under both insufficient-demand and sufficient-demand scenarios. This result indicates that expert rank selection is mainly determined by customers’ sensitivity to expert rank and the wage differentiation level within the industry, rather than by the overall market demand scale. This finding simplifies the expert recruitment decision and suggests that enterprises should align expert selection with long-term strategic positioning.
(2) When market demand is insufficient, increased customer sensitivity to expert rank encourages enterprises to provide higher-quality expert services. In this case, enterprises tend to hire higher-ranked experts and set longer service duration to improve customers’ perceived utility. Meanwhile, firms can charge a higher service price to compensate for the increased expert salary cost and improve profitability.
(3) When market demand is sufficient, enterprises should adopt a “low-margin, high-volume” strategy. As demand increases, firms can reduce service duration and lower service prices to attract and serve more customers. This strategy improves service throughput and enables enterprises to achieve higher profits than in the insufficient-demand scenario.

The theoretical contribution of this study lies in extending the application of queueing theory to expert service operations by endogenizing expert rank and service time within a unified analytical framework. Unlike traditional service queueing models that mainly focus on waiting time, service rate, or pricing decisions, this study examines how expert rank and service duration jointly affect customer utility and further derives the optimal decisions regarding expert selection and service duration. Moreover, by integrating the customer market and the expert labor market, this study captures the interaction among customer demand, expert compensation, and firm operational decisions. It also analyzes firm strategies under different demand scenarios, thereby providing meaningful managerial insights for expert service enterprises.

### 7.2 Managerial implications

The findings of this study provide several managerial implications for expert service enterprises and related industry stakeholders.

(1) Expert service enterprises should regard expert rank selection as a long-term strategic decision rather than a short-term response to demand fluctuations. Firms should determine the appropriate expert rank mainly according to customers’ sensitivity to expert rank and the wage differentiation level in the expert labor market. In practice, enterprises should avoid frequently adjusting their expert recruitment standards merely in response to temporary changes in market demand. This issue has not been fully addressed in existing studies [7–10].
(2) Expert service enterprises should adopt differentiated service and pricing strategies under different demand conditions. When demand is insufficient, firms should enhance customers’ perceived utility by hiring higher-ranked experts and providing longer service duration. In this situation, a higher service price can be used to compensate for the increased expert salary cost. In contrast, when demand is sufficient, firms should implement a “low-margin, high-volume” strategy characterized by shorter service duration and lower service price, which helps firms serve more customers and improve profitability. This result further extends the findings of Zhan et al. [34].
(3) Managers should carefully balance the customer market and the expert labor market. Hiring experts whose rank is too low may reduce customers’ willingness to join the service system, whereas hiring experts whose rank is too high may impose excessive salary costs on the enterprise. Therefore, when designing their service systems, expert service enterprises should jointly consider customer utility, waiting cost, service price, service duration, and expert compensation.
(4) It is important to improve the transparency and standardization of expert service markets. In industries such as healthcare, consulting, auditing, and maintenance services, clear expert qualification standards and reasonable service pricing mechanisms can help reduce inefficient resource allocation. In particular, demand-guidance mechanisms may help alleviate congestion for high-ranked experts while improving the utilization of lower-ranked experts.

### 7.3 Future research and limitations

This study also has several limitations that provide opportunities for future research. First, the model assumes that expert rank perfectly reflects expert capability and that rank information is transparent to customers. However, in practice, information asymmetry may exist, and expert rank may not fully represent actual service capability. Future studies can consider the effect of information asymmetry on customer perceived utility and enterprise decision-making. Second, this paper considers a single-server M/M/1 queueing system. Future research may extend the model to multi-server systems or service systems with multiple experts of different ranks. Third, this study focuses on a single expert service enterprise. Future research can further examine competition among multiple expert service enterprises and analyze how competitive interactions affect expert recruitment, pricing, and service duration decisions. Finally, empirical studies can be conducted in industries such as healthcare, consulting, auditing, and maintenance services to validate the theoretical findings of this paper.

## Supporting information

S1 Data Minimal numerical data underlying the figures reported in the manuscript. (XLSX)

S1 Appendix Proofs of propositions and corollaries. (PDF)
