# Supplementary material for: The optimal operation decisions of expert service enterprises based on queuing system
Source: PLoS One. 2026 Oct 30;21(10):e0360850. doi: 10.1371/journal.pone.0360850 (PMC13722332; doi:10.1371/journal.pone.0360850)
Supplement: Supplementary file 1 [file pone.0360850.s002.pdf]

S1 Appendix. Proofs of propositions and corollaries.

A Appendix

A.1 Proof of Proposition 1

The proof of Proposition 1 can be intuitively derived from Equations (10)–(13); therefore, it is omitted here for brevity.

A.2 Proof of Proposition 2

The proof of Proposition 2 can be intuitively derived from Equations (14) and (15); therefore, it is omitted here for brevity.

A.3 Proof of Proposition 3

The proof of Proposition 3 can be intuitively derived from Equations (18); therefore, it is omitted here for brevity.

A.4 Proof of Proposition 4

The proof of Proposition 4 can be intuitively derived from Equations (19) and (20); therefore, it is omitted here for brevity.

A.5 Proof of Corollary 1.

By taking the first-order derivative of $t^*_{ins} = \frac{1}{\Lambda}\left(1 - \sqrt{\frac{c}{\epsilon(r_0+1)-2\sqrt{\epsilon\alpha}}}\right)$ with respect to ϵ, we obtain

$$\frac{\partial t^*_{ins}}{\partial \epsilon} = \frac{c}{2\Lambda}\left(\frac{c}{\epsilon\left(r_0+1\right)-2\sqrt{\epsilon\alpha}}\right)^{-\frac{1}{2}} \frac{\left(r_0+1\right)-\sqrt{\alpha/\epsilon}}{\left[\epsilon\left(r_0+1\right)-2\sqrt{\epsilon\alpha}\right]^2}.$$

It can be seen that the sign of $\frac{\partial t^*_{ins}}{\partial \epsilon}$ is determined by $(r_0+1)-\sqrt{\frac{\alpha}{\epsilon}}$. When $\epsilon \geq \frac{\alpha}{(r_0+1)^2}$, we have $\frac{\partial t^*_{ins}}{\partial \epsilon} \geq 0$. When $\epsilon \leq \frac{\alpha}{(r_0+1)^2}$, we have $\frac{\partial t^*_{ins}}{\partial \epsilon} \leq 0$.

Meanwhile, it is necessary to ensure that the denominator term satisfies $\epsilon\left(r_0+1\right) - 2\sqrt{\epsilon\alpha} > 0$. Thus, we have $\epsilon > \frac{4\alpha}{(r_0+1)^2} > \frac{\alpha}{(r_0+1)^2}$. Since t^*_{ins} cannot be negative, it is also necessary to ensure that $1 - \sqrt{\frac{c}{\epsilon(r_0+1)-2\sqrt{\epsilon\alpha}}} \geq 0$. Solving this condition yields $\epsilon \geq \frac{\sqrt{\alpha}+\sqrt{\alpha+c(r_0+1)}}{r_0+1}$.

Once $\epsilon < \frac{\sqrt{\alpha}+\sqrt{\alpha+c(r_0+1)}}{r_0+1}$, customers' sensitivity in the service market is relatively low. In addition, because the market demand scale is small, any service duration provided by experts would lead to losses for the service enterprise. Under this circumstance, the service enterprise will exit the market.

Therefore, there exists

$$\epsilon_m = \max\left\{\frac{4\alpha}{\left(r_0+1\right)^2}, \frac{\sqrt{\alpha}+\sqrt{\alpha+c\left(r_0+1\right)}}{r_0+1}\right\}.$$

When $\epsilon \in (\epsilon_m, \alpha)$, we have $\frac{\partial t^*_{ins}}{\partial \epsilon} \geq 0$. Thus, when the market demand scale is small, the target customers should satisfy the condition $\epsilon \in (\epsilon_m, \alpha)$, where $\epsilon_m = \max\left\{\frac{4\alpha}{(r_0+1)^2}, \frac{\sqrt{\alpha}+\sqrt{\alpha+c(r_0+1)}}{r_0+1}\right\}$.

A.6 Proof of Corollary 2

When the demand scale is insufficient, the optimal price is given by

$$P^*_{ins}\left(r^*_{ins},t\right) = V_0 + \epsilon\left(r_0+1\right)t - t\sqrt{\epsilon\alpha} - \frac{ct}{1-\Lambda t}.$$

Taking the first-order and second-order derivatives of $P^*_{\text{ins}}\left(r^*_{\text{ins}},t\right)$ with respect to t, we obtain $\frac{\partial P^*_{ins}(r^*_{ins},t)}{\partial t} = \epsilon\left(r_0+1\right) - \sqrt{\epsilon\alpha} - \frac{c}{(1-\Lambda t)^2}$, and $\frac{\partial^2 P^*_{ins}(r^*_{ins},t)}{\partial t^2} = -\frac{c}{(1-\Lambda t)^4} < 0$.

By setting $\frac{\partial P^*_{ins}(r^*_{ins},t)}{\partial t} = 0$, we obtain $t^*_p = \frac{1}{\Lambda}\left(1-\sqrt{\frac{c}{\epsilon(r_0+1)-\sqrt{\epsilon\alpha}}}\right)$. Since $\frac{\partial^2 P^*_{ins}(r^*_{ins},t)}{\partial t^2} < 0$, $P^*_{ins}\left(r^*_{ins},t\right)$ reaches its maximum at $t=t^*_p$, and $P^*_{ins}\left(r^*_{ins},t\right)$ is increasing over the interval $\left(\underline{t},t^*_p\right)$.

Because$t^*_{ins} = \frac{1}{\Lambda}\left(1-\sqrt{\frac{c}{\epsilon(r_0+1)-2\sqrt{\epsilon\alpha}}}\right)$, we have $t^*_{ins} < t^*_p$. Therefore, when $t = t^*_{ins}$, $\frac{\partial P^*_{ins}(r^*_{ins},t)}{\partial t} > 0$.

Moreover, when $\epsilon \in (\epsilon_m, \alpha)$, we have $\frac{\partial t^*_{ins}}{\partial \epsilon} \geq 0$. Thus, according to the chain rule, when $t = t^*_{ins}$, we obtain

$$\frac{\partial P^*_{ins}\left(r^*,t\right)}{\partial \epsilon} = \frac{\partial P^*_{ins}\left(r^*_{ins},t\right)}{\partial t}\frac{\partial t^*_{ins}}{\partial \epsilon} \geq 0,$$

for $\epsilon \in (\epsilon_m, \alpha)$. According to the envelope theorem, by taking the derivative of $\pi^*_{ins}\left(P^*_{ins}, r^*_{ins}, t^*_{ins}\right)$ with respect to ϵ, we obtain $\frac{\partial \pi^*_{ins}}{\partial \epsilon} = \left(r_0+1-\sqrt{\frac{\alpha}{\epsilon}}\right)t^*_{ins}$.

Since $\epsilon_m = \max\left\{\frac{4\alpha}{(r_0+1)^2}, \frac{\sqrt{\alpha}+\sqrt{\alpha+c(r_0+1)}}{r_0+1}\right\}$, we have $r_0+1-\sqrt{\frac{\alpha}{\epsilon}} > 0$. Therefore, it must hold that $\frac{\partial \pi^*_{ins}}{\partial \epsilon} > 0$.

A.7 Proof of Corollary 3

It can be shown that

$$P^*_{suf}\left(r^*_{suf},t^*_{suf}\right) = V_0 + \epsilon\left(r_0+1\right)\underline{t} - \underline{t}\sqrt{\epsilon\alpha} - \sqrt{c\underline{t}\left[V_0+\epsilon\left(r_0+1\right)\underline{t} - 2\underline{t}\sqrt{\epsilon\alpha}\right]}.$$

Equivalently,

$$P^*_{suf}\left(r^*_{suf},t^*_{suf}\right) = V_0 + \epsilon\left(r_0+1\right)\underline{t} - 2\underline{t}\sqrt{\epsilon\alpha} - \sqrt{c\underline{t}\left[V_0+\epsilon\left(r_0+1\right)\underline{t} - 2\underline{t}\sqrt{\epsilon\alpha}\right]} + \underline{t}\sqrt{\epsilon\alpha}.$$

Taking the derivative with respect to ϵ, we obtain

$$\frac{\partial P^*_{suf}\left(r^*_{suf},t^*_{suf}\right)}{\partial \epsilon} = \underline{t}\left[\left(1-\sqrt{\frac{c\underline{t}}{V_0+\epsilon\left(r_0+1\right)\underline{t}-2\underline{t}\sqrt{\epsilon\alpha}}}\right)\left(\left(r_0+1\right)-\sqrt{\frac{\alpha}{\epsilon}}\right)+\frac{1}{2}\sqrt{\frac{\alpha}{\epsilon}}\right].$$

When $\epsilon \in \left[\frac{\alpha}{(r_0+1)^2}, \alpha\right]$, we have $(r_0+1)-\sqrt{\frac{\alpha}{\epsilon}} > 0$. Therefore, $\frac{\partial P^*_{suf}}{\partial \epsilon} > 0$. When $\epsilon \in \left(0, \frac{\alpha}{(r_0+1)^2}\right)$, we have $(r_0+1)-\sqrt{\frac{\alpha}{\epsilon}} < 0$. In this case, it is necessary to examine the magnitude of

$$1-\sqrt{\frac{c\underline{t}}{V_0+\epsilon\left(r_0+1\right)\underline{t}-2\underline{t}\sqrt{\epsilon\alpha}}},$$

which depends on c. Let $\bar{c} = \frac{V_0}{\underline{t}} + \epsilon\left(r_0+1\right) - 2\sqrt{\epsilon\alpha}$. As $c \to \bar{c}_1$, we have $1-\sqrt{\frac{c\underline{t}}{V_0+\epsilon(r_0+1)\underline{t}-2\underline{t}\sqrt{\epsilon\alpha}}} \to 0$. Thus, $\lim_{c\to\bar{c}} \frac{\partial P^*_{suf}}{\partial \epsilon} > 0$.

When $c \to 0$, we have $1 - \sqrt{\frac{c\underline{t}}{V_0+\epsilon(r_0+1)\underline{t}-2\underline{t}\sqrt{\epsilon\alpha}}} \to 1$. Therefore, there must exist a threshold $\bar{\epsilon}$ such that $\frac{\partial P^*_{suf}}{\partial \epsilon} = 0$, where $\bar{\epsilon} = \max_{\epsilon \in \left(0, \frac{\alpha}{(r_0+1)^2}\right)} \left\{\epsilon \,\middle|\, \frac{\partial P^*_{suf}}{\partial \epsilon} = 0\right\}$. Hence, for $\epsilon \in (0, \bar{\epsilon}]$, we have $\frac{\partial P^*_{suf}}{\partial \epsilon} \leq 0$.

A.8 Proof of Corollary 4

The effective customer arrival rate is given by

$$\lambda^*_{suf}\left(P^*_{suf}, r^*_{suf}, t^*_{suf}\right) = \frac{1}{\underline{t}} - \sqrt{\frac{c}{\underline{t}\left[V_0 + \epsilon\left(r_0+1\right)\underline{t} - 2\underline{t}\sqrt{\epsilon\alpha}\right]}}.$$

Taking the derivative of λ^*_{suf} with respect to ϵ, we obtain

$$\frac{\partial \lambda^*_{suf}}{\partial \epsilon} = \frac{\underline{t}\sqrt{c}}{2\left[V_0 + \epsilon\left(r_0+1\right)\underline{t} - 2\underline{t}\sqrt{\epsilon\alpha}\right]^{3/2}}\left[\left(r_0+1\right)\underline{t} - \underline{t}\sqrt{\frac{\alpha}{\epsilon}}\right].$$

When $\frac{\partial \lambda^*_{suf}}{\partial \epsilon} = 0$, we obtain $\epsilon = \frac{\alpha}{(r_0+1)^2}$. When $\frac{\partial \lambda^*_{suf}}{\partial \epsilon} > 0$, we have $\epsilon > \frac{\alpha}{(r_0+1)^2}$.

Since $\epsilon < \alpha$, the interval over which λ^*_{suf} increases with ϵ is $\epsilon \in \left[\frac{\alpha}{(r_0+1)^2}, \alpha\right)$. When $\frac{\partial \lambda^*_{suf}}{\partial \epsilon} < 0$, we have $\epsilon < \frac{\alpha}{(r_0+1)^2}$. Therefore, the interval over which λ^*_{suf} decreases with ϵ is $\epsilon \in \left(0, \frac{\alpha}{(r_0+1)^2}\right)$. The firm's optimal profit function is given by

$$\pi^*_{suf}\left(P^*_{suf}, r^*_{suf}, t^*_{suf}\right) = \left[\sqrt{\frac{V_0 + \epsilon\left(r_0+1\right)\underline{t} - 2\underline{t}\sqrt{\epsilon\alpha}}{\underline{t}}} - \sqrt{c}\right]^2.$$

Taking the derivative of π^*_{suf} with respect to ϵ, we obtain

$$\frac{\partial \pi^*_{suf}}{\partial \epsilon} = \frac{1}{\underline{t}}\left[1 - \sqrt{\frac{c\underline{t}}{V_0 + \epsilon\left(r_0+1\right)\underline{t} - 2\underline{t}\sqrt{\epsilon\alpha}}}\right]\left[\left(r_0+1\right)\underline{t} - \underline{t}\sqrt{\frac{\alpha}{\epsilon}}\right].$$

Setting $\frac{\partial \pi^*_{suf}}{\partial \epsilon} = 0$, gives $\epsilon = \frac{\alpha}{(r_0+1)^2}$. When $\frac{\partial \pi^*_{suf}}{\partial \epsilon} > 0$, we have $\epsilon > \frac{\alpha}{(r_0+1)^2}$. Since $\epsilon < \alpha$, the interval over which π^*_{suf} increases with ϵ is $\epsilon \in \left[\frac{\alpha}{(r_0+1)^2}, \alpha\right)$.

When $\frac{\partial \pi^*_{suf}}{\partial \epsilon} < 0$, we have $\epsilon < \frac{\alpha}{(r_0+1)^2}$. Therefore, the interval over which π^*_{suf} decreases with ϵ is $\epsilon \in \left(0, \frac{\alpha}{(r_0+1)^2}\right)$.

A.9 Proof of Corollary 5

i. When demand is sufficient, the price is given by

$$P^*_{suf}\left(r^*_{suf}, t\right) = V_0 + \epsilon\left(r_0+1\right)t - t\sqrt{\epsilon\alpha} - \sqrt{ct\left[V_0 + \epsilon\left(r_0+1\right)t - 2t\sqrt{\epsilon\alpha}\right]}.$$

When demand is insufficient, the optimal price is given by

$$P^*_{ins}\left(r^*_{ins}, t\right) = V_0 + \epsilon\left(r_0+1\right)t - t\sqrt{\epsilon\alpha} - \frac{ct}{1-\Lambda t}.$$

Taking the first-order and second-order derivatives of P^*_{ins} with respect to t, we obtain $\frac{\partial P^*_{ins}}{\partial t} = \epsilon\left(r_0+1\right) - \sqrt{\epsilon\alpha} - \frac{c}{(1-\Lambda t)^2}$, and $\frac{\partial^2 P^*_{ins}}{\partial t^2} = -\frac{c}{(1-\Lambda t)^4} < 0$. By setting

$\frac{\partial P_{ins}^*}{\partial t} = 0$, we obtain $t_p^* = \frac{1}{\Lambda}\left(1-\sqrt{\frac{c}{\epsilon(r_0+1)-\sqrt{\epsilon\alpha}}}\right)$. Since $\frac{\partial^2 P_{ins}^*}{\partial t^2} < 0$, P_{ins}^* reaches its maximum at $t = t_p^*$, and P_{ins}^* is increasing over the interval $\left(\underline{t}, t_p^*\right)$. Because $t_{ins}^* = \frac{1}{\Lambda}\left(1-\sqrt{\frac{c}{\epsilon(r_0+1)-2\sqrt{\epsilon\alpha}}}\right)$, we have $t_{ins}^* < t_p^*$.

Therefore, $P_{ins}^*\left(r_{ins}^*, t_{ins}^*\right) < P_{ins}^*\left(r_{ins}^*, t_p^*\right)$. Moreover, since $\underline{t} < t_{ins}^*$, we have $P_{ins}^*\left(r_{ins}^*, \underline{t}\right) < P_{ins}^*\left(r_{ins}^*, t_{ins}^*\right)$.

Next, we examine how P_{ins}^* changes with Λ. Clearly, P_{ins}^* decreases as Λ increases. Since $\Lambda \leq \lambda_{suf}^*\left(r_{suf}^*, \underline{t}\right) = \frac{1}{\underline{t}} - \sqrt{\frac{c}{\underline{t}\left[V_0+\epsilon(r_0+1)\underline{t}-2\underline{t}\sqrt{\epsilon\alpha}\right]}}$, P_{ins}^* reaches its minimum when $\Lambda = \lambda_{suf}^*$. Thus, when $\Lambda = \lambda_{suf}^*$ and $t = \underline{t}$, it must hold that $P_{ins}^*\left(r_{ins}^*, \underline{t}\right) < P_{ins}^*\left(r_{ins}^*, t_{ins}^*\right)$.

When $\Lambda = \lambda_{suf}^*\left(r_{suf}^*, \underline{t}\right)$, and $t = \underline{t}$, we have $P_{ins}^*\left(r_{ins}^*, \underline{t}\right) = V_0 + \epsilon\left(r_0+1\right)\underline{t} - \underline{t}\sqrt{\epsilon\alpha} - \sqrt{c\underline{t}\left[V_0+\epsilon\left(r_0+1\right)\underline{t}-2\underline{t}\sqrt{\epsilon\alpha}\right]}$. Thus, $P_{ins}^*\left(r_{ins}^*, \underline{t}\right) = P_{suf}^*\left(r^*, \underline{t}\right)$. Therefore, it must hold that $P_{suf}^*\left(r^*, \underline{t}\right) < P_{ins}^*\left(r^*, t_{ins}^*\right)$.

ii. For the profit, the service enterprise's profit under insufficient demand is given by

$$\pi_{ins}^*\left(P_{ins}^*, r_{ins}^*, t\right) = \left[V_0 + \epsilon\left(r_0+1\right)t - 2t\sqrt{\epsilon\alpha} - \frac{ct}{1-\Lambda t}\right]\Lambda.$$

Taking the first-order and second-order derivatives with respect to Λ, we obtain

$$\frac{\partial \pi_{ins}^*}{\partial \Lambda} = V_0 + \epsilon\left(r_0+1\right)t - 2t\sqrt{\epsilon\alpha} - \frac{ct}{(1-\Lambda t)^2},$$

and $\frac{\partial^2 \pi_{ins}^*}{\partial \Lambda^2} = -\frac{2\Lambda(1-\Lambda t)}{(1-\Lambda t)^4} < 0$. By setting $\frac{\partial \pi_{ins}^*}{\partial \Lambda} = 0$, we obtain $\Lambda_{ins}^* = \frac{1}{t} - \sqrt{\frac{c}{t\left[V_0+\epsilon(r_0+1)t-2t\sqrt{\epsilon\alpha}\right]}} = \lambda_{suf}^*$.

Since $\frac{\partial^2 \pi_{ins}^*}{\partial \Lambda^2} < 0$, we know that when $\Lambda \leq \Lambda_{ins}^* \pi_{ins}^*$ increases with Λ, and reaches its maximum at $\Lambda = \Lambda_{ins}^*$. Substituting $\Lambda = \Lambda_{ins}^* = \frac{1}{t} - \sqrt{\frac{c}{t\left[V_0+\epsilon(r_0+1)t-2t\sqrt{\epsilon\alpha}\right]}}$ into π_{ins}^*, we obtain

$$\max_{\Lambda=\Lambda_{ins}^*} \pi_{ins}^*\left(P_{ins}^*, r_{ins}^*, t\right) = \left[\sqrt{\frac{V_0 + \epsilon\left(r_0+1\right)t - 2t\sqrt{\epsilon\alpha}}{t}} - \sqrt{c}\right]^2.$$

That is, the maximum value of π_{ins}^* has the same form as π_{suf}^*. Under this form, according to $\frac{\partial \pi_{suf}^*}{\partial t} = -\left[\sqrt{\frac{V_0+\epsilon(r_0+1)t-2t\sqrt{\epsilon\alpha}}{t}} - \sqrt{c}\right]\left[\sqrt{\frac{V_0+\epsilon(r_0+1)t-2t\sqrt{\epsilon\alpha}}{t}}\right]^{-\frac{1}{2}} \frac{V_0}{t^2} < 0$, the profit decreases as t increases. Since $\underline{t} < t_{ins}^*$, we have

$$\max_{\Lambda=\Lambda_{ins}^*} \pi_{ins}^*\left(P_{ins}^*, r_{ins}^*, t_{ins}^*\right) < \pi_{suf}^*\left(P_{suf}^*, r_{suf}^*, \underline{t}\right).$$

Therefore, it must hold that

$$\pi_{ins}^*\left(P_{ins}^*, r_{ins}^*, t_{ins}^*\right) < \pi_{suf}^*\left(P_{suf}^*, r_{suf}^*, \underline{t}\right).$$
